# Gene expression profiling of skeletal myogenesis in human embryonic stem cells reveals a potential cascade of transcription factors regulating stages of myogenesis, including quiescent/activated satellite cell-like gene expression

**DOI:** 10.1371/journal.pone.0222946

**Published:** 2019-09-27

**Authors:** Michael Shelton, Morten Ritso, Jun Liu, Daniel O’Neil, Avetik Kocharyan, Michael A. Rudnicki, William L. Stanford, Ilona S. Skerjanc, Alexandre Blais

**Affiliations:** 1 Department of Biochemistry, Microbiology, and Immunology, Faculty of Medicine, University of Ottawa, Ottawa, Ontario, Canada; 2 Sprott Center for Stem Cell Research, Ottawa Hospital Research Institute, Regenerative Medicine Program, Ottawa, Ontario, Canada; 3 Department of Cellular and Molecular Medicine, Faculty of Medicine, University of Ottawa, Ottawa, Ontario, Canada; 4 Ottawa Institute of Systems Biology, University of Ottawa, Ottawa, Ontario, Canada; 5 Department of Medicine, Faculty of Medicine, University of Ottawa, Ottawa, Ontario, Canada; University of Minnesota Medical School, UNITED STATES

## Abstract

Human embryonic stem cell (hESC)-derived skeletal muscle progenitors (SMP)—defined as PAX7-expressing cells with myogenic potential—can provide an abundant source of donor material for muscle stem cell therapy. As *in vitro* myogenesis is decoupled from *in vivo* timing and 3D-embryo structure, it is important to characterize what stage or type of muscle is modeled in culture. Here, gene expression profiling is analyzed in hESCs over a 50 day skeletal myogenesis protocol and compared to datasets of other hESC-derived skeletal muscle and adult murine satellite cells. Furthermore, day 2 cultures differentiated with high or lower concentrations of CHIR99021, a GSK3A/GSK3B inhibitor, were contrasted. Expression profiling of the 50 day time course identified successively expressed gene subsets involved in mesoderm/paraxial mesoderm induction, somitogenesis, and skeletal muscle commitment/formation which could be regulated by a putative cascade of transcription factors. Initiating differentiation with higher CHIR99021 concentrations significantly increased expression of MSGN1 and TGFB-superfamily genes, notably NODAL, resulting in enhanced paraxial mesoderm and reduced ectoderm/neuronal gene expression. Comparison to adult satellite cells revealed that genes expressed in 50-day cultures correlated better with those expressed by quiescent or early activated satellite cells, which have the greatest therapeutic potential. Day 50 cultures were similar to other hESC-derived skeletal muscle and both expressed known and novel SMP surface proteins. Overall, a putative cascade of transcription factors has been identified which regulates four stages of myogenesis. Subsets of these factors were upregulated by high CHIR99021 or their binding sites were significantly over-represented during SMP activation, ranging from quiescent to late-activated stages. This analysis serves as a resource to further study the progression of *in vitro* skeletal myogenesis and could be mined to identify novel markers of pluripotent-derived SMPs or regulatory transcription/growth factors. Finally, 50-day hESC-derived SMPs appear similar to quiescent/early activated satellite cells, suggesting they possess therapeutic potential.

## Introduction

Stem cell therapy is the delivery of healthy donor cells to repair damaged or diseased tissue. In skeletal muscle, the most well-studied type of skeletal muscle progenitor (SMP) is the muscles’ own resident stem cell: satellite cells, characterized by the expression of PAX7 [1,2]. Adult murine satellite cells can be isolated with relatively established surface markers, such as ITGA7 and VCAM1 [3–5], and satellite cells’ quiescent status can be distinguished by established gene expression markers, like SPRY1 and JAG1 [6–8]. The signaling cascades from quiescent to activated satellite cell, and from myoblast to myotube, are also the subject of extensive study in the adult muscle environment, reviewed in [9,10]. However, cultured satellite cells quickly lose their quiescent phenotype and enter the less therapeutically ideal activated state, marked by expression of myogenic regulatory factors (MRFs)[2,11]. It is therefore difficult to obtain large quantities of donor satellite cells which may be required to treat a patient’s entire musculature.

Alternative types of SMPs can be derived from more proliferative source material, like SMPs derived from the directed differentiation of human pluripotent stem cells (hPSC)[12,13]. It is also possible to isolate SMPs from the fetal satellite cell compartment [14], or from the milieu of extensively dividing progenitors during primary and secondary myogenesis [15–18]. As stem cell therapy will ultimately require human cells, however, these latter options become difficult to obtain in appreciable quantities. Thus, the directed differentiation of hPSCs circumvents these practical issues.

Recent years have seen a rise in the number of studies that direct the differentiation of appreciable proportions of hPSCs into skeletal muscle [12,13,19–25]. Previously we developed a protocol that initiated differentiation with 10 μM CHIR99021, while other protocols that also begin with CHIR99021 mostly do so with 3 μM or less [19,20,22–25]. Our protocol is capable of leading approximately 90% of cells into the skeletal muscle lineage after 50 days in culture: 43 ± 4% of cells were PAX7^+^ skeletal muscle progenitors (SMP), surrounded by myosin heavy chain (MYH) expressing myocytes and small myotubes that comprised another 47 ± 3% of the population [12,13].

These hPSC-derived SMPs remain poorly characterized relative to *in vivo* myogenic cells and especially to adult satellite cells. As hPSCs are detached from the architecture and timing of *in utero* development, it is unclear how closely the directed *in vitro* myogenesis of hPSCs follows our understanding of *in vivo* myogenesis. For example, if the *in vitro* cascades of gene expression are similar to the embryo from pluripotency through to myotubes, or if some developmental stages are bypassed in culture, as may be the case with the transgenic-driven myogenesis of human embryonic stem cells (hESCs) via MYOD1 over-expression [26,27].

It is also critical to establish what stage of pre-natal myogenesis may ultimately be represented by day 50 of *in vitro* hESC differentiation, be it primary myogenesis, secondary myogenesis wherein myoblasts show greater propensity for fusion and true satellite cells first appear [28,29], or a different profile altogether. If directed hESC differentiation does generate SMPs that share characteristics with satellite cells, it would be important to understand if these cells also undergo a similar process of quiescence and activation. This may have implications for day 50 cells’ *in vitro* expansion, or for their efficacy upon transplant into the muscle of mouse models.

A recent study suggests that day 50 hPSC-derived SMPs are functionally similar to late embryonic muscle [18]. However, the gene expression analysis performed by Hicks *et al*. was limited to NCAM1^+^ sorted cells, which are limited at *in vitro* myogenesis and engraftment, so no information could be gathered concerning the cultures as a whole. Unbiased gene expression profiling of 50-day cells may therefore provide a broader resource to study all myogenic populations present in culture.

Last is the question of unwanted tissue lineages that may arise during *in vitro* hPSC differentiation; generating the greatest proportion of myogenic cells possible would be most cost effective for stem cell therapy, and the absence of off-target cell types would be required to limit unwanted side-effects upon the cultures’ *in vivo* transplantation. Gene expression profiling could identify genes that demarcate off-target lineages; expression profiling and knowledge of these lineages’ development can suggest surface markers to negatively select them via cell sorting, or suggest additional small molecule treatments to curb their emergence.

These facts all underscore the importance of directly studying hPSC-derived myogenic cultures in a thorough unbiased manner. Therefore, this study aims to use gene expression profiling to characterize SMPs and the type of muscle derived from hESCs via the previously published 50-day *in vitro* differentiation protocol [12,13]. Four main clusters of genes with similar expression patterns were identified over the 50-day time courses that are related to skeletal myogenesis. Comparisons between these genes and satellite cell datasets, or comparison with myogenic cultures derived via a different directed differentiation protocol, identified several shared key transitional regulators between hESC-derived cultures and quiescent/early activated satellite cells. A number of cell surface receptors shared between hESC-derived cultures and either early or late activated satellite cells were also identified, as were a number of receptors that were unique to day 50 cultures; these unique cell surface proteins may provide novel avenues with which to isolate or demarcate subsets of myogenic populations present at day 50 of *in vitro* hESC myogenesis.

## Results and discussion

### Expression pattern similarity identifies co-regulated developmental gene sets important for *in vitro* mesoderm induction and skeletal myogenesis

Gene expression profiling was used at various time points of the *in vitro* skeletal myogenesis protocol in order to characterize the cells’ progression in greater detail [12]. Briefly, the differentiation of pluripotent hESCs was initiated by culturing cells in E6 medium supplemented with high concentrations (7.5 or 10 μM) of CHIR99021 for 2 days. Cells were then cultured in E6 without CHIR99021 until day 12, at which point medium was changed to StemPro-34 supplemented with 5 ng/mL FGF2 until day 20. Cells were returned to E6 medium from days 20 to 35, and then cultured in N2-ITS medium from days 35 to 50.

**Fig 1:**
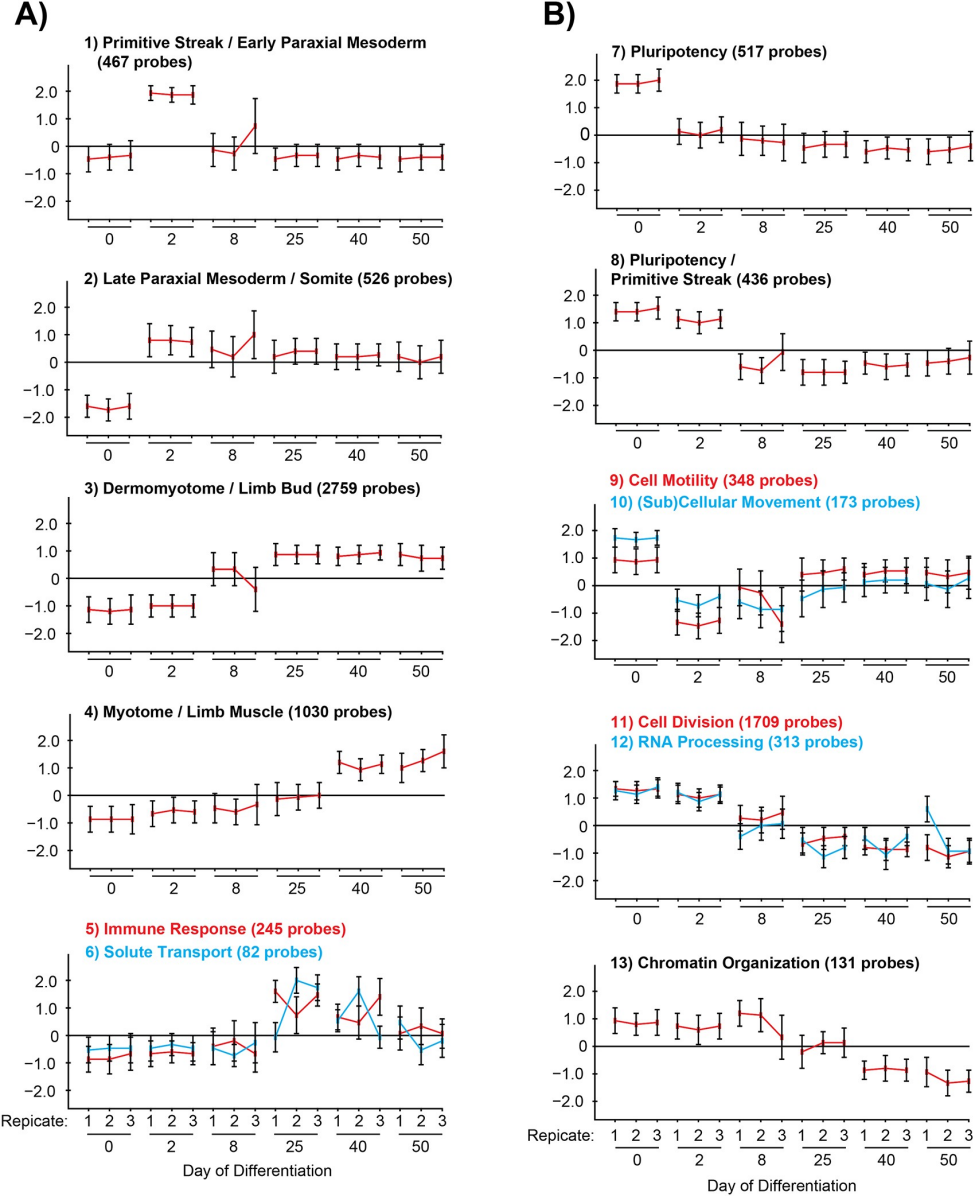
Gene clustering identifies up- and down-regulated groups that control development, pluripotency, and cell division. Pluripotent H9 hESCs were differentiated for 50 days as previously described [12], beginning with high (7.5 μM or 10 μM) CHIR99021 concentrations. mRNA samples from the indicated time points were subjected to gene expression profiling. Gene clustering based on expression profiles generated 13 clusters of similarly expressed probes across the differentiation time course. Similarly patterned clusters were manually grouped for further analysis, and clusters were divided into (A) genes whose expression increased with differentiation, and (B) genes whose expression decreased. A full list of gene identities within each cluster can be found in S1 Table.

Genes sharing a similar expression profile across the 50 day time course initiated with high CHIR99201 were clustered using the CLICK clustering algorithm [30], resulting in 6 groups of genes that showed increasing expression as differentiation proceeded (Fig 1A), and 7 groups that showed decreasing expression (Fig 1B)(S1 Table). ToppFun function enrichment analysis was used to identify significantly enriched Gene Ontology (GO) categories within each cluster [31], and also to identify significantly enriched Progenitor Cell Biology Consortium (PCBC) Co-expression Atlas datasets for select clusters [32]. GO categories revealed that clusters 1 through 4 show a progression through primitive streak and paraxial mesoderm genes, to somitic and supportive developmental genes, to myogenic commitment and structural constituents of contractile muscle (Table 1).

**Table 1:** Gene Ontology characterization of gene clusters shows progressive development through the expected stages of skeletal myogenesis, and progressive drop of pluripotency and cell cycling genes expected throughout differentiation.

Cluster	# of Genes	GO: Biological Process	q-value	Genes / Families	Soleimani (/ 294)	Lilja (/ 1302)
		GO:0009952—Anterior/posterior specification	5.0 E-11	FGF8, MSGN1, WNT8A	4 (1.36%) 5.76 E-01	24 (1.84%) 8.68 E-02
1	275	GO:0061053—Somite development	3.6 E-05	DLL3, FOXC1, LFNG		
		GO:0007498—Mesoderm Development	1.8 E-02	MIXL1, T, TBX6		
		GO:0048598—Embryonic morphogenesis	2.5 E-23	HOXs, PDGFRA, PAX3	13 (4.42%) 5.73 E-03	54 (4.15%) 1.64 E-07
2	395	GO:0030509—BMP signaling pathway	8.6 E-06	BMP1/4/6/7, MSX1/2		
		GO:0016055—WNT signaling pathway	4.2 E-04	DKK1, RSPO3, WNT3A/5A/5B		
		GO:0030198—ECM organization	2.1 E-23	COLs, LAMs, MMPs	81 (27.6%) 5.33 E-17	237 (18.2%) 3.23 E-19
3	2067	GO:0061061—Muscle structure development	1.7 E-12	DMD, MEOX1/2, NCAM1		
		GO:0006811—Ion transport	9.2 E-03	CACNs, KCNs, SLCs		
		GO:0006936—Muscle contraction	9.7 E-27	CHRNs, MYH/Ls, TNNC/Ts	40 (13.6%) 6.34 E-13	68 (5.22%) 1.65 E-03
4	736	GO:0014706—Striated muscle development	7.6 E-24	MSTN, MRFs, PAX7		
		GO:0014902—Myotube differentiation	6.8 E-07	MEF2C, MYOD1, MYOG		
		GO:0007155—Cell adhesion	1.2 E-04	CDH7, ITGA1/B2, PECAM1	7 (2.38%) 9.66 E-02	22 (1.69%) 1.43 E-01
5 + 6	267	GO:0006955—Immune response	2.6 E-04	C1Qs, FCGRs, IL6ST/7		
		GO:0006811—Ion transport	6.1 E-02	CCLs, CYBB, SLCs		
		Ratio Stem Cell vs. Induced-Mesoderm:			6 (2.04%) 3.56 E-02	21 (1.61%) 5.73 E-01
7	331	Top 500 Ranked by Relative-expression	8.9 E-32	FGF2, NANOG, SOX2		
		PCBC_ratio_SC_vs_MESO-5_500				
		Ratio Stem Cell vs. Induced-Mesoderm:			4 (1.36%) 6.66 E-01	13 (1.00%) 9.67 E-01
8	310	Top 500 Ranked by Relative-expression	7.2 E-08	ENHO, FGF19, POU5F1		
		PCBC_ratio_SC_vs_MESO-5_500				
		GO:0006928—Cell / subcellular movement	2.2 E-06	KIFs, LMNA, PODXL/2	19 (6.46%) 8.55 E-06	54 (4.15%) 3.15 E-07
9 + 10	406	GO:0048729—Tissue morphogenesis	1.9 E-05	GLI2, KDR, PDGFA/B		
		GO:0048870 –Cell motility	2.1 E-04	ARC, EPCAM, MMPs		
		GO:0007049—Cell cycle	4.9 E-40	CDCs, CDKs, KIFs	11 (3.74%) 9.93 E-01	70 (5.36%) 9.93 E-01
11 + 12	1394	GO:0006260—DNA replication	5.9 E-25	BRAC1/2, MCMs, PRIM1/2		
		GO:0006396—RNA processing	9.6 E-15	PRMTs, RRPs, SRSFs		
		GO:0050000—Chromosome localization	4.4 E-07	CENPs, CEP55, KIFs	0 (0%) 0	5 (0.38%) 7.52 E-01
13	97	GO:0051276—Chromosome organization	3.1 E-06	NUF2, PRMT6, REC8		
		GO:0071103—DNA conformation change	4.1 E-02	ERN2, HIST1Hs, HIST2Hs		

The gene clusters generated in Fig 1A and 1B were passed through ToppFun functional enrichment analysis (p < 0.05, FDR < 0.05). Representative categories of GO:Biological Process were selected for each cluster, as well as representative genes or gene families within each GO category. Where clusters 7 and 8 contained little or no significant GO categories, these clusters were significantly enriched for genes more highly expressed in pluripotent stem cells relative to induced mesoderm, according to the Progenitor Cell Biology Consortium (PCBC) expression atlas [32]. Genes from each cluster were compared against a list of genes known to be differentially expressed in murine myoblasts with Pax3- or Pax7-over expression [33], and genes whose promoters are directly bound by Pax7 [34]. The percentage of either lists’ genes that were represented within each cluster was determined, and the hypergeometric probability of each result was calculated (p(X) ≥ n). Clusters 2–4, and 9 + 10 were notably enriched for a significant number of Pax7 regulated genes.

The “Primitive Streak / Early Paraxial Mesoderm” cluster represented the transient expression of expected mesoderm genes including T, TBX6, and MSGN1 [35–37]. The onset of NOTCH-related segmentation genes DLL3, HES7, and LFNG by day 2 of differentiation was consistent with other *in vitro* myogenesis protocols, wherein somite-related gene expression can be detected starting from 1.75–2.25 days [25,38]. Analysis of promoter sequences of the genes in this cluster revealed a significant over-representation of transcription factor binding sites (TFBS) for the downstream effectors of CHIR99021 and WNT signaling, LEF and TCF (Table 2)(S2A and S2B Table). The TAL1::TCF3 TFBS—which is the most significantly similar consensus sequence to that of MSGN1 [39]–was also present. The POU5F1 (OCT4) motif was also significantly enriched; while POU5F1 is an important factor for maintaining pluripotency, persistent POU5F1 protein levels are also required for T (Brachyury) and mesoderm induction *in vitro* and *in vivo* [40–42].

**Table 2:** Transcription factor binding site analysis of gene clusters identified several known developmental myogenesis factors, suggesting co-identified and previously unstudied factors may also play important roles.

Cluster	# of Genes	Transcription Factor Binding Sites (Z-score)
1	275	LEF1 (21.2), TCF7L2 (19.9), TCF7 (15.7), HOXA3 (14.1), MSGN1 (10.1), SMAD3 (8.9), POU5F1 (8.1), PBX1 (7.4), PPARG (6.9), RUNX1 (6.0), E2F1 (5.8), MYF6 (5.6), HOXA5 (5.3)
2	395	HOXA5 (16.8), LEF1 (16.1), TCF7L2 (15.1), PRRX2 (12.2), HNF1A (12.2), *TCF7 (11*.*6)*, *ARID3A (11*.*1)*, FOXA2 (9.7), FOXA2 (9.4), MYBL1 (9.0), FOXJ1 (8.6), SOX21 (8.5), SPIB (8.4), SOX5 (7.2), *T (6*.*6)*, FOXD3 (6.2), GATA5 (5.7), OSR1 (5.5), SOX5 (5.3), FOXD1 (5.3), *MSGN1 (5*.*2)*, ZBTB12 (5.0)
3	2067	*HOXA5 (28*.*2)*, ARID3A (27.2), FOXD3 (23.0), FOXA2 (22.6), FOXQ1 (20.5), SOX5/6 (20.1), CEBPA (18.4), PRRX1/2 (17.3), *FOXD1 (15*.*9)*, SOX2 (12.2), SPIB (9.9), MEF2A (7.3), HNF1A (6.0), HLF (5.3)
4	736	KLF4 (17.4), MEF2C/D (17.1), *INSM1 (12*.*6)*, MRFs (12.5), MYC (9.4), *EBF1 (9*.*2)*, NR3C1 (9.1), MYCN (8.1), *NHLH1 (7*.*4)*, *PLAG1 (7*.*1)*, *PPARG (5*.*9)*
5 + 6	267	ARID3A (23.3), PRRX2 (18.6), HOXA5 (18.4), FOXD3 (14.8), SOX5 (14.5), NR2F1 (11.3), FOXA2 (11.2), NR3C1 (10.8), POU5F1 (10.8), CEBPA (9.7), FOXD1 (8.0), SPIB (7.0), HNF1A (6.4), ELF5 (5.2)
7	331	KLF4 (14.2), SOX2 (11.2), POU5F1 (10.7), ZEB1 (5.1)
8	310	NHLH1 (10.3), INSM1 (7.3), PPARG (7.1), EBF1 (5.6)
9 + 10	406	PRRX2 (11.4), POU5F1 (11.2), HOXA5 (10.7), ARID3A (8.3), PBX1 (8.3), SOX2 (8.1), FOXA2 (7.7), FOXD1 (7.3), FOXQ1 (6.2), RUNX1 (5.3)
11 + 12	1394	PPARG (15.6), E2F1 (14.9), MYC (13.0), MYCN (12.4), TP53 (9.4), KLF4 (7.4), ZEB1 (5.5)
13	97	NR3C1 (8.1), NR2F1 (6.3)

Gene clusters from Fig 1A and 1B were also analyzed to identify significant TFBSs. Significantly enriched motifs were listed for each cluster group (Z-score > 5). Only motifs whose parent transcription factor were represented in the clustering analysis were listed. Motifs whose parent transcription factor was contained within the same cluster are underlined. For clusters 1–4 only: motifs whose parent transcription factor fell within the previous cluster are italicized. The significantly enriched TAL1::TCF3 motifs in the two earliest clusters have been manually attributed to MSGN1, owing to the two sites’ similarity [39]. The complete list of TFBS results can be found in S2A–S2L Table.

The “Late Paraxial Mesoderm / Somite” and “Dermomyotome / Limb Bud” clusters contained transcription factors and markers that may denote the differentiation of paraxial mesoderm into muscle progenitors and myoblasts, including genes that mark the somite and limb muscle progenitors that migrate from the hypaxial dermomyotome PAX3, MEF2A, MET, and DMD [43,44](Table 1). Of note, PAX3, BMP, and WNT signaling genes peak in the early stages of the differentiation protocol but do not fall back down to baseline day 0 levels, suggesting that after the initial burst in expression, lower levels of activity in these pathways are sufficient for continuation of the myogenic program.

The “Myotome / Limb Muscle” group largely represented genes involved in the terminal differentiation of skeletal muscle, including the myogenic regulatory transcription factors (MRF) MYF5, MYOD1, MYOG and MYF6, and genes of the contractile apparatus: myosin light and heavy chains, troponins, Z-disc proteins, sarcoglycans, and acetylcholine receptors [45]. Even though PAX7 is considered a marker of muscle progenitors rather than terminally differentiated muscle, its expression profile could not be separated from the MRFs, likely due to the lack of resolution between the days 8 and 25 used in this study. Other studies of *in vitro* hESC myogenesis—that differentiate cells with 3 μM CHIR99021 for 4 days followed by Notch inhibition via DAPT for 8 days—distinguish notable MYOG upregulation approximately 2 days after PAX7 [24].

TFBS analysis identified a number of known developmental or myogenic regulators in the “Dermomyotome / Limb Bud” and “Myotome / Limb Muscle” clusters such as HOXs, CEPB, MEF2, and MRFs (Table 2)(S2E and S2F Table), validating this approach. Therefore, co-enriched TFBSs that are less well studied in the context of skeletal muscle development—like ARID3A, KLF4, and INSM1—may also play important roles and are worthy of further study. In order to determine whether PAX3 or PAX7 may be transcriptionally active, we took advantage of experimentally determined indirect PAX3 or PAX7 target gene lists from murine myoblasts over-expressing either transcription factor for 2 days [33], and direct PAX7 targets in murine ESC-derived muscle progenitors after 3 days of induced PAX7 expression [34]. The greatest portion of experimentally determined PAX3 and PAX7 target genes fell under the third cluster with 24.6% and 15.3% of the Soleimani *et al*. and Lilja *et al*. lists being represented, respectively (Table 1). It was interesting to observe that the majority of PAX7-bound genes from the Lilja *et al*. dataset began to elevate their expression at day 8, at a time when PAX3 expression is already near its maximum levels and PAX7 expression is on the rise. Of the 237 PAX7-bound genes in the third cluster, only 19 were also shared with the Soleimani *et al*. dataset, including EFEMP1, EGFR, MEIS1, PDGFD, and PITX2. These genes may play an important role throughout somite and myogenic development, and PAX7 may be partly responsible for their continued regulation in the context of more advanced development stages of skeletal muscle. PITX2 for example plays a role in regulating dermomyotome development with PAX3, and PITX2 is also involved in regulating adult satellite cell proliferation [46,47]. EGFR signaling is important in adult satellite cell differentiation and polarity [48,49], it is highly expressed in fetal muscle [50], and EGF supplementation increased hPSC myogenic differentiation efficiency [21]. The identification of PITX2 and EGFR in our dataset suggests that other genes identified by this method could be important as well. Therefore, results of clustering and TFBS analysis have identified a putative cascade of transcription factors that bind either to genes of their own cluster or neighboring clusters, potentially orchestrating early events of myogenesis.

The “Immune Response” and “Solute Transport” clusters were small groups of genes with transient changes in expression—enriched with GO categories related to the immune system—that only appear elevated from the day 25 time point before gradually falling. This may reflect a response to the StemPro-34 media or supplement introduced from days 12 to 20 of the protocol, which are formulated with glucocorticoids to support the culture and differentiation of hematopoietic or immune-system cells [51].

Clusters that showed a downward trend over time were enriched for genes and GO categories expected to drop as pluripotent cells differentiate; for example, “Cell Division,” “RNA Processing,” and “Chromatin Organization” categories. The expected decrease in expression for genes related to cell proliferation correlated with previous findings, wherein cell numbers taper off logarithmically over the 50 day differentiation [12]. Clusters 7 and 8 did not have any statistically significant-enriched GO categories, however, these clusters were significantly enriched for genes of the PCBC Coexpression Atlas dataset wherein mesoderm was induced from hESCs; indeed, the pluripotency factors NANOG and SOX2 belong to the rapidly down-regulated “Pluripotency” cluster, and POU5F1 belonged to the delayed down-regulated “Pluripotency / Primitive Streak” cluster.

**Fig 2:**
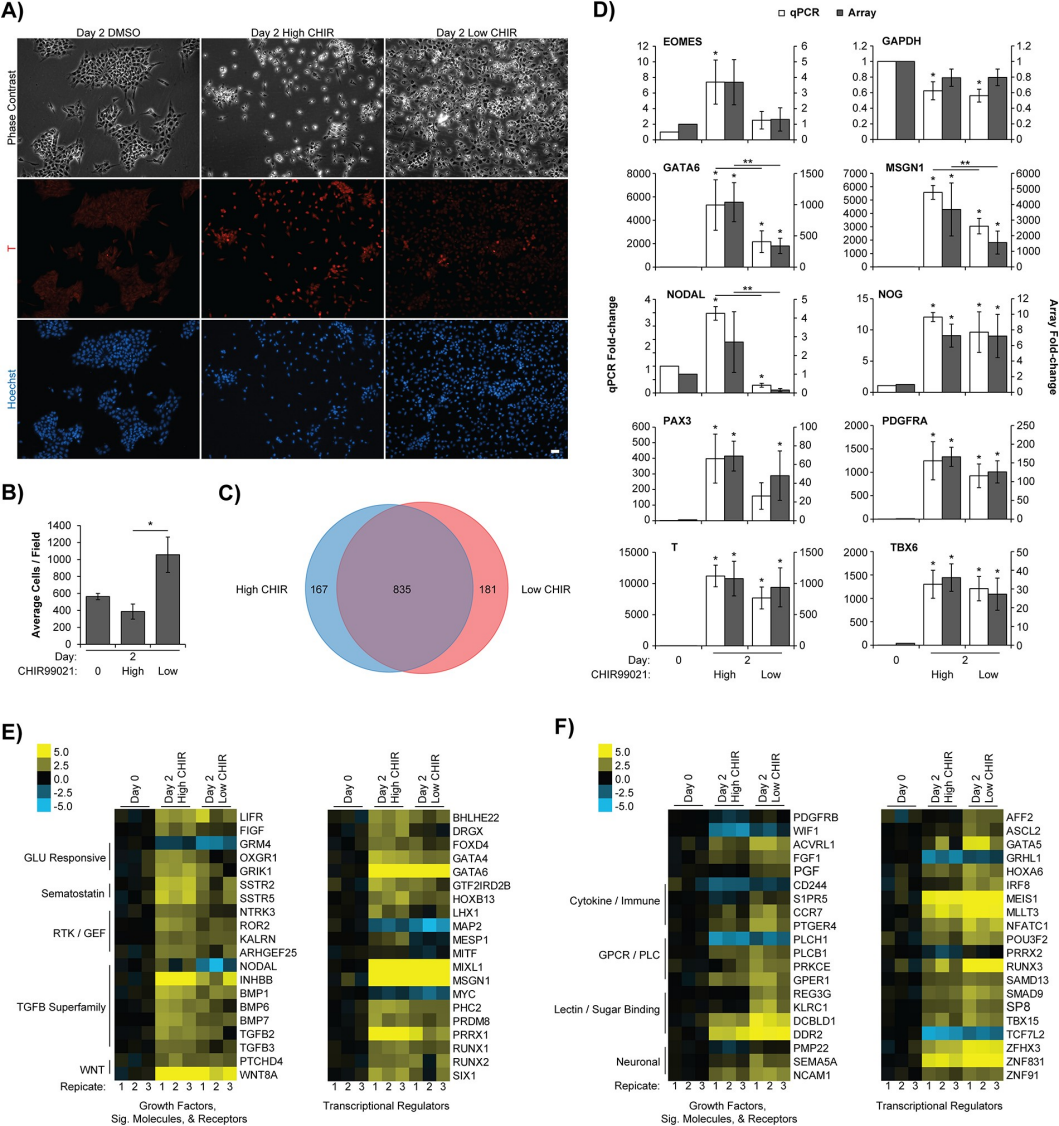
The highest tolerable CHIR99021 concentrations induce significantly more paraxial mesoderm gene expression than 3 μM. Pluripotent H9 hESCs were differentiated with either 7.5 μM (High) or 3 μM (Low) CHIR99021 for 2 days, and with DMSO as a vehicle control. (A) At day 2, cells were fixed and stained with antibodies against T (Red) and Hoechst dye (Blue) to visualize cell nuclei (Scale bar = 20 μm). (B) Volocity 6.0 software was used to count the average number of cells and the proportion of T^+^ cells with high or low CHIR99021 treatment from 10 randomly chosen fields. High CHIR99021-treated cultures contained significantly fewer total cells than low CHIR99021-treated (n = 3, **p < 0.05) according to one-way ANOVA with post-hoc Tukey test (n = 3, p < 0.05). (C) Day 2 mRNA samples from high and low CHIR99021 treated cultures were subjected to gene expression profiling. Expander 7.0 t-test identified 835 probes that were significantly upregulated at day 2 in both the high and low CHIR99021 conditions, relative to day 0 (n = 3, p < 0.05, FDR < 0.05). High and low CHIR99021 conditions showed 167 and 181 uniquely expressed probes, respectively. (D) Select genes identified in (C) were validated by qPCR. The qPCR results were expressed as a fold-change on the primary Y-axis, and the microarray results on the secondary Y-axis, both relative to day 0 control (n = 3, *p < 0.05). Several genes—including MSGN1 and NODAL—were significantly more expressed in high CHIR99021-treated cultures according to Expander 7.0 t-test of the microarray, and one-way ANOVA with post-hoc Tukey test of the qPCR (n = 3, **p < 0.05). Expander 7.0 t-test was also used to identify 67 and 88 additional probes that showed significantly differential expression between a direct comparison of high and low CHIR99021 conditions (n = 3, p < 0.05). The resulting genes—along with the 167 & 181 uniquely expressed probes from (C)—were classified using PANTHER to identify growth factors, signaling molecules, receptors, and transcriptional regulators. Heat maps were generated for the top 20 differentially expressed genes with (E) more expression in high CHIR99021-treatment, and (F) more expression in low CHIR99021-treatment. Individual replicates are shown for each condition. Replicate expression values were standardized to the average of the day 0 triplicates.

The remaining clusters 9 & 10 were significantly enriched for numerous GO categories related to motility and cell-cell contacts. A decrease in cell-cell contact-related genes might be expected to occur immediately following CHIR99021 treatment, as CHIR99021-treated day 2 cells appear morphologically more segregated than tightly packed pluripotent colonies (Fig 2A)[12,13]. CHIR99021 and WNT signaling support epithelial to mesenchymal transition (EMT) in ESCs, increasing cell velocity in cells expressing T in comparison to other cells present in the culture [52]. Thus, our results suggest that the average motility of the culture is transiently reduced on day 2, followed by a gradual partial recovery.

Overall, the examination of gene expression pattern similarity has identified co-regulated gene sets that represent the developmental stages of *in vitro* myogenesis, from mesoderm and somitogenesis to dermomyotome, myotome and limb muscle.

### CHIR99021 concentration-dependent differences in expression of T/MSGN1 and NODAL/TGFB signaling genes

Application of 3 μM or lower CHIR99021 to human pluripotent stem cells is one of the more prevalent methods to induce mesoderm [38,52–58], especially for downstream skeletal myogenesis [20,22–24]. However, the Shelton *et al*. study showed that higher levels of CHIR99021 led to greater expression of mesoderm and paraxial mesoderm genes T and MSGN1, respectively, and that only high levels of CHIR99021 induced PAX3 expression and PAX3^+^ cells by day 8 of differentiation [12]. Therefore, both high and low CHIR99021-induced day 2 mesoderm were compared by gene expression analysis to determine what differences existed between the two approaches, and how that might explain the more efficient paraxial mesoderm induction with high CHIR99021.

Confirming previous results, high (7.5 μM) CHIR99021 treatment led to significantly more prominent T staining (Fig 2A), corresponding to 73 ± 6% T^+^ cells compared to low CHIR99021 treatment with 21 ± 11% T^+^ cells out of total cells (n = 3, p < 0.05 by one-way ANOVA with post-hoc Tukey test). Furthermore, cell numbers remained relatively stagnant between days 0 to 2 in high CHIR99021-treated cultures and total cell number was significantly lower relative to low (3 μM) CHIR99021 and DMSO control (Fig 2B). Beginning differentiation with relatively higher hESC densities correlated with more neuronal differentiation [59].

mRNA samples from high and low CHIR99021-treated day 2 cultures were also subjected to gene expression profiling to elucidate an underlying mechanism that might explain the differing effectiveness between both approaches. There were 835 probes significantly elevated in both approaches by at least 4-fold relative to day 0 control, with 167 and 181 probes uniquely elevated in high or low CHIR99021 treatment, respectively (Fig 2C). A direct comparison of high and low CHIR99021-treated day 2 cultures showed the paraxial mesoderm marker MSGN1 was expressed at significantly higher levels in high CHIR99021-treated cells; qPCR for MSGN1 validated these findings (Fig 2D).

Studies have shown that 5–10 μM CHIR99021 concentrations lead to more pronounced T expression or staining—compared to 2–3 μM—within 2 days post-treatment in hPSCs [60,61]. Others have shown that hESCs differentiated with 7.5 μM CHIR99021 for 2 days leads to notably higher expression of mesoderm markers T and PDGFRA compared to 3 μM [57]. In their study, only 3 μM CHIR99021 treated hESCs showed expression of endoderm markers FOXA2 and SOX17. Therefore, low concentrations of CHIR99021 may be permissible for mesendoderm formation in hPSCs while high concentrations restrict endoderm and lead to greater proportions of mesoderm derivatives, including MSGN1-expressing paraxial mesoderm.

Genes significantly elevated only in high CHIR99021 treatment were enriched for TGFB receptor binding (Table 3), while genes elevated only with low CHIR99021 showed significant enrichment for the category of neuronal differentiation (Table 4). Interestingly, gene set enrichment analysis showed that genes induced by EGF signaling were also more expressed with high CHIR99021, including AREG, EGR1/2/3/4, and DUSP2/5 (S1 Fig). High levels of TGFB signaling have been shown to block ectoderm specification from pluripotent cells, and to specify anterior primitive streak-like mesoderm from which the paraxial mesoderm is derived [38]. In contrast, having low to moderate levels of TGFB signaling favors the developmental pathway of lateral plate mesoderm. The reduced expression of TGFB signaling genes with low compared to high CHIR99021-treament may therefore result in lower repression of ectoderm or neuron formation, and less paraxial mesoderm specification.

**Table 3:** TGFB signaling Gene Ontology categories are significantly enriched among genes more highly expressed is high CHIR99021 conditions.

GO Category	q-value (E-02)	Genes in Query (/ 139)	Example Genes
TGFB-receptor binding	0.88	5	BMP6, BMP7, GDNF, TGFB2, TGFB3
Epithelial cell differentiation	0.80	16	ABCB1, BMP6, BMP7, CES1, DMBT1, FSHR, GDNF, HOXB13, HPS1, LHX1, NKX6-1, NTRK3, OCA2, SIX1, TGFB2, WNT4
Embryonic skeletal system morphogenesis	0.97	7	BMP7, HYAL1, LHX1, RUNX2, SIX1, TGFB2, TGFB3
Cellular response to endogenous stimulus	2.08	22	BMP6, BMP7, FSHR, GDNF, IGFBP5, LHX1, NKX6-1, RUNX1, RUNX2, SSTR2, SSTR5, TGFB2, TGFB3, WNT4
Response to endogenous stimulus	3.37	26	ATP2A1, BMP6, BMP7, FSHR, GDNF, HOXB13, IGFBP5, KALRN, LHX1, NKX6-1, NTRK3, RUNX1, RUNX2, SSTR2, SSTR5, TGFB2, TGFB3, WNT4
Embryonic cranial skeletal morphogenesis	4.26	5	LHX1, RUNX2, SIX1, TGFB2, TGFB3

Probes from Fig 2C were passed through ToppFun functional enrichment analysis (p < 0.05, FDR < 0.05). A complete list of significant GO:Molecular Function and GO:Biological Process categories and select representative genes were tabulated.

**Table 4:** Neuronal differentiation Gene Ontology categories are significantly enriched among genes more highly expressed is low CHIR99021 conditions.

GO Category	q-value (E-02)	Genes in Query (/ 158)	Example Genes
Neuron differentiation	2.10	26	CDH4, COL25A1, DGKG, EPB41L3, FZD1, ISPD, NIN, NRXN3, POU3F2, PTCH1, RAPH1, RNF165, RUNX3, SARM1, SATB2, SLITRK5, TCF4
Positive regulation of developmental process	2.46	25	ACVRL1, CCR7, CDH4, FGF1, GPER1, NIN, NRXN3, PLCB1, POU3F2, PTCH1, PTGER4, RAG1, SLITRK5, SMAD9, TCF4, TMEM119
Generation of neurons	3.55	27	CDH4, COL25A1, DGKG, EPB41L3, FZD1, GPER1, ISPD, NIN, NRXN3, POU3F2, PTCH1, RAPH1, RNF165, RUNX3, SARM1, SATB2, SLITRK5, TCF4
Positive regulation of lipid metabolic process	4.41	8	ACSL6, CCR7, DAB2, FGF1, IRS1, PPARA, PRKCE, SIRT4

Probes from Fig 2C were passed through ToppFun functional enrichment analysis (p < 0.05, FDR < 0.05). A complete list of significant GO:Molecular Function and GO:Biological Process categories and select representative genes were tabulated.

The implication of TGFB as a discriminator between high- and low-CHIR99021 protocols was again highlighted by a different type of analysis, where we used PANTHER classification to identify the specific signaling pathways and transcriptional regulators in an unbiased manner. The 167 and 181 significantly expressed probes from Fig 2C were organized using PANTHER, as were additional differentially expressed probes from a direct comparison of high and low CHIR99021-treated cultures. The growth factors, signaling molecules, receptors, and transcriptional regulators more highly expressed with high CHIR99021 were identified (Fig 2E), as were the genes with higher expression in low CHIR99021 cultures (Fig 2F).

One of the starkest differences between protocols appeared to be the TGFB superfamily, and specifically NODAL signaling family members. NODAL signaling, along with WNT, are essential for *in vitro* primitive streak induction [62] and WNT signaling regulates NODAL expression [63,64]. The data presented in Fig 2 may indicate a dose-dependent mechanism, whereby high CHIR99021 better upregulated NODAL than low CHIR99021 treatment, and thus, led to a more efficient induction of primitive streak-like cells *in vitro*. Known NODAL target genes CER1 and LHX1 were also more highly expressed with high CHIR99021 [65,66], supporting the notion that NODAL signaling is more active in high CHIR99021-treated cells.

Beyond the day 2 *in vitro* induction of mesoderm in hPSCs, however, high TGFB or NODAL signaling directs this mesoderm into the endodermal lineage, while chemical inhibition of TGFBR1, ACVR1B, and ACVR1C receptors with A8301 or SB431542 enhances somite-like mesoderm [23,25,38]. These facts notwithstanding, the 50-day differentiation protocol presented here yields largely myogenic cultures without the addition of pharmacological TGFB superfamily inhibitors. As mentioned above, high levels of WNT signaling restrict endoderm formation [57], possibly by regulating TGFB-superfamily inhibitor genes CER1, CHRD, and NOG [67]. CER1 directly binds and inhibits NODAL and BMP proteins [68], and we found its expression to be elevated in high CHIR99021-treated cells. In fact, gene expression analysis identified CER1 as the most similarly-expressed gene to NODAL over the entire gene expression profiling dataset (S3 Table). Therefore, endogenous mechanisms to inhibit NODAL and BMP may already be active in day 2 cultures, supporting the efficient development of somite-like mesoderm from the paraxial mesoderm *in vitro*. A recent study has also implicated endogenously secreted CER1 as having profound effects on CHIR99021-based mesoderm induction in hPSCs [69].

Therefore, congruent with previous studies that show higher levels of CHIR99021 promote mesodermal induction [57,60,61], this study shows that higher levels of CHIR99021 induced significantly greater expression of MSGN1 in hESCs compared to lower CHIR99021. Greater upregulation of TGFB superfamily genes—specifically NODAL signaling and CER1—may provide the underlying mechanism for these observations.

### Day 50 hESC-derived skeletal muscle cultures share more similar gene expression with quiescent or early activated than with late activated satellite cells

One goal of *in vitro* skeletal myogenesis is to generate satellite cell-like SMPs that could be used in stem cell therapy, both to repair injured muscle and replenish the resident muscle stem cell population. In order for SMPs to engraft efficiently, PAX7^+^ SMPs should not yet express MYOD1, as expression of MYOD1 may indicate that cells have differentiated too far to self-replenish the progenitor population in muscle [11].

It was previously shown that 43 ± 4% of cells stain positive for PAX7 at day 50 of differentiation [12]. Here, co-immunostaining allowed us to discern a significant proportion of PAX7-expressing cells that express no detectable MYOD1 protein (S2 Fig). Immunostaining for PAX7 and KI67 was also performed, which revealed a sizable number of PAX7^+^ cells that show no detectable KI67. Further, there was a strong inverse correlation between the levels of PAX7 and those of KI67 or MYOD1 (S2 Fig). Taken together, these observations suggest that hESC-derived myogenic cultures may contain quiescent-like PAX7^+^ cells.

**Fig 3:**
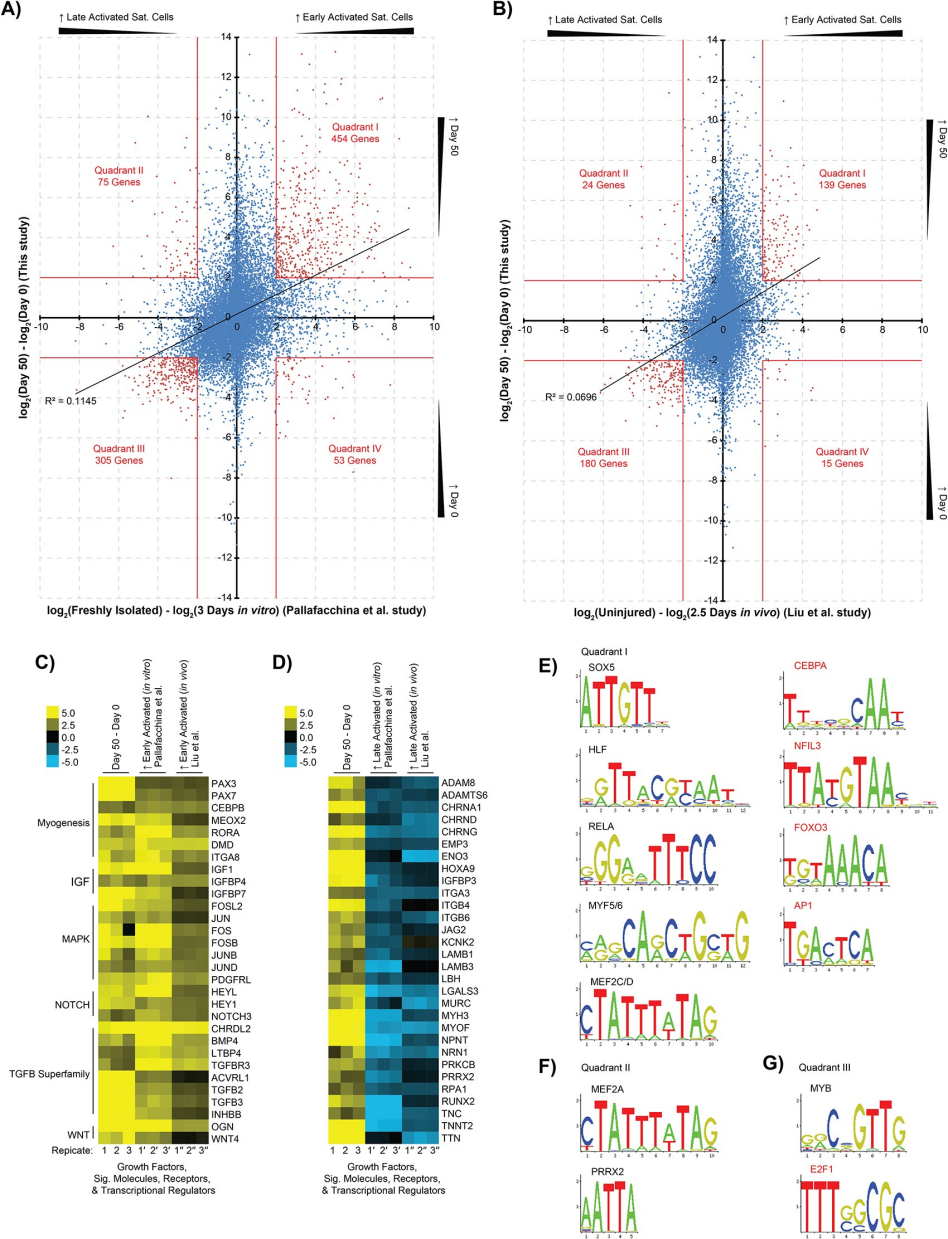
Day 50 cultures share elevated FOS/JUN, NOTCH, and TGFB-signaling with early activated satellite cells. The mean day 50 expression of individual genes were standardized to the mean of day 0 samples, while the mean expression of genes from (A) *in vitro*-cultured [70] or (B) *in vivo*-derived [8] early activated satellite cells were standardized to the mean of their respective late activated satellite cell samples (n = 3). Gene lists were generated from the four plot quadrants of (A) and (B) given an absolute 4-fold change cut-off. Genes from (C) quadrant I, and (D) quadrant II were classified using PANTHER to identify growth factors, signaling molecules, receptors, and transcriptional regulators. Day 50 cultures shared more genes in common with early- than late-activated satellite cells, including FOS/JUN, NOTCH, and TGFB-signaling genes, along with other notable genes BMP4, CEBPB, DMD, IGF1, and PAX7. Day 50 cultures and late activated satellite cells shared more structural and ion channel genes, such as LAMB1/3, MYH3, TTN, and the Cholinergic Receptor Nicotinic (CHRN) gene gamily. Quadrant gene lists were also analyzed to identify significantly enriched TFBS. Only motifs whose parent transcription factor was contained within the same quadrant were considered, given a 2-fold change cut-off. Significant motifs from *in vitro*-cultured satellite cells are shown for (E) quadrant I, (F) quadrant II, and (G) quadrant III; quadrant IV yielded no significant results. Motifs that are highlighted red are enriched in both *in vitro*-cultured (A) and *in vivo*-derived (B) satellite cell quadrants.

To further explore similarities and differences between day 50 cultures and quiescent or activated satellite cell populations, we compared our expression data with two other datasets. Briefly, the *in vitro* activation experiment by Pallafacchina *et al*. (Fig 3A)[70], extracted RNA from satellite cells isolated from adult Pax3^GFP/+^ mice, either immediately after sorting—termed early activated—or following 3 days of *in vitro* culture-induced activation—termed late activated. Obtaining satellite cells from muscle can require 5 hours or less of processing, during which time the cells enter an early activation phenotype that has been shown to be distinct from the true quiescent G0-phase phenotype found *in vivo* in the satellite cell niche of uninjured adult muscle [71,72]. The *in vivo* activation experiment by Liu *et al*. (Fig 3B)[8], extracted RNA from isolated satellite cells of adult Pax7^CreER/+^/ROSA26^eYFP/+^ mice, either from uninjured muscle—termed early activated—or muscle that was injured with barium chloride 2.5 days prior to sorting—termed late activated.

A total of 13 874 or 14 498 genes were paired between the hESC array and the Pallafacchina *et al*. *in vitro* or Liu *et al*. *in vivo* activation experiments, respectively. The expression values of each gene from the hESC array were plotted against their counterpart from the *in vitro* or *in vivo* activated satellite cells (Fig 3A and 3B, respectively), where a positive correlation was seen between day 50 cultures and early activated satellite cells. For further analysis, the expression plots were divided into four quadrants based on an arbitrarily chosen 4-fold cutoff. Quadrant I—genes more highly expressed at day 50 and in early activated satellite cells—was enriched with GO categories related to muscle structure development. Key genes PAX7 and PAX3 displayed similar trends but did not meet the 4-fold cutoff of quadrant I with *in vivo* activation, showing 3.5- and 3-fold higher expression in early activated satellite cells, respectively. Given the established importance of PAX7 and PAX3 in maintaining quiescence, however, these genes were considered part of quadrant I for further analysis of the *in vivo* activation dataset.

Quadrant II—genes more highly expressed at day 50 and in late activated satellite cells—was enriched in extracellular matrix organization or muscle contraction genes (Fig 3A and 3B). Unsurprisingly, genes more highly expressed in late activated satellite cells and day 0 pluripotent cells—quadrant III—were enriched for GO categories related to the cell cycle.

Notable signaling pathways or transcription factors that might play key roles in regulating the gene subsets shared by day 50 cultures and early activated satellite cells were also investigated (Fig 3C), to provide clues to the presence of a persistent PAX7^+^ population and relatively few large myotubes in these cultures [12]. PANTHER classification of quadrant I genes showed important embryonic myogenesis transcription factors MEOX2 and PAX3. Analysis also identified the CEBPB transcription factor, which can repress MYOD1 function and reduces myoblasts’ capacity to differentiate or fuse [73], the dystrophin gene (DMD), and surface markers ICAM1, VCAM1, and ITGA8. ICAM1 and VCAM1 are known to mark embryonic-derived SMPs or adult satellite cells [3,4,74], but no role has been described for ITGA8 in developing skeletal muscle or satellite cells. Interestingly, the expression profiling shows a 6- to 22-fold increase in ITGA8 across the three datasets, suggesting it plays an important role at this stage. Additional PANTHER classification for growth factors and signaling molecules identified genes involved in maintaining the adult satellite cell progenitor state or quiescence: namely, NOTCH signaling [75–78], TGFB signaling [79], and several FOS/JUN-related genes downstream in MAPK signaling [80–82]. NOTCH signaling enhances the PAX7^+^/MRF^-^ muscle progenitor phenotype [2,83].

Relatively fewer transcription factors were identified in quadrant II: day 50 cultures were found to share only RUNX2 and PRRX2 with *in vitro* activated satellite cells (Fig 3D). No shared transcription factors were identified between day 50 cultures and quadrant II of *in vivo* activated satellite cells given the 4-fold change cut-off. RUNX2 is normally expressed in C2C12 myoblasts and activated satellite cells [84,85], but it does not appear to be required for skeletal myogenesis [86]. PRRX2 plays a role in limb development [87]; and may co-regulate target gene expression with SOX8, which prevents satellite cell differentiation [88,89]. Quadrant II also contained surface proteins ITGA3, ITGB4, and ITGB6 in both, *in vitro*, and *in vivo* datasets, respectively. ITGA3 supports myoblast adhesion and fusion [90], while the roles of ITGB4 and ITGB6 in satellite cells are less well defined. ITGB4 may be a MYOD1 target when satellite cells are cultured *in vitro* [91], and ITGB6 protein rapidly accumulates in murine skeletal muscle after freeze injury but with no established function [92]. These surface proteins may provide a method of isolating the late activated SMP populations from hESC-derived myogenic cultures as a negative selection to remove cells that have differentiated beyond the ideal PAX7^+^/MRF^-^ therapeutic stage.

All quadrant gene lists were then analyzed within their promoter regions for significantly enriched TFBSs to help establish a functional role for the transcription factors found within each list. TFBS enrichment analysis of quadrant I genes showed the AP1 FOS/JUN target motif, CEBP leucine zipper, and FOXO forkhead box motifs were significantly enriched with day 50 cultures and early activated satellite cells (Fig 3E)(S2M and S2N Table). Various FOS/JUN proteins and CEBPB are responsible for regulating MYOD1 activity or stability [73,80–82]. FOXO3 maintains satellite cell quiescence, potentially through activation of NOTCH3 [93]. Further, the binding motifs for the MRFs, MEF2C/D, and SOX box transcription factors were significantly enriched with day 50 cultures and early activated Pax3^GFP/+^ satellite cells. Results showed that SOX5 and SOX6 were expressed in both day 50 cultures and early activated satellite cells. SOX6 represses slow-type fiber formation during embryonic myogenesis and may repress sarcomeric or contraction-related gene expression—including CHRNG and TNNT2—and downregulate transcription factors like PROX1, MYOD1, and MYOG [94,95]. SOX5 likewise has an established role in embryonic myogenesis [96]; however, its role in satellite cells has not been studied. Interestingly, transcription factor binding sites for HLF, RELA, and NFIL3 were also identified in a significant number of quadrant I genes, suggesting that the transcription factors that bind these sites may also play important roles in early activated satellite cells. Finally, key TFBSs representing CEBPA, HLF, and SOX5/6 that were identified within genes shared between day 50 cultures and early activated satellite cells were also represented in the “Dermomyotome / Limb Bud” cluster from previous analysis of the hESC differentiation time course (Table 2)(S2E Table).

Considering the genes in quadrant II with *in vitro* late activated satellite cells, their promoters were significantly enriched for the MEF2A MADS box and PRRX2 homeodomain binding motifs (Fig 3F)(S2O and S2P Table). The results for PRRX2’s relatively short AATTA consensus sequence may also represent genes bound by other homeodomain proteins—which are known to generally recognize highly related sequences [97]—including MEOX2, PAX3, PAX7 [33] and the PRRX2 homolog PRRX1. Although PAX3 and PAX7 were considered part of quadrant I, a significant number of genes within quadrant II have been shown to be bound by PAX7, including RUNX2 and CAVIN4 [34]. MEF2A is well established in muscle regeneration, and its expression is expected to spike 3 days post-activation in satellite cells [43]. Interestingly, other MEF2 family members elevated in day 50 cultures were more highly expressed in early activated satellite cells relative to their *in vitro* activated counterparts; quadrant I contained MEF2C (18-fold) and MEF2D (2-fold). TFBSs for factors involved in DNA binding and cell proliferation were linked to day 0 and late activated satellite cell cultures as expected (Fig 3G)(S2Q and S2R Table).

Overall, the expression profiling of day 50 cultures revealed a more prominent representation of FOS/JUN, NOTCH, and TGFB signaling pathways—as well as the downstream transcriptional effectors of these pathways—that correlated more with early activated rather than late activated satellite cells.

**Fig 4:**
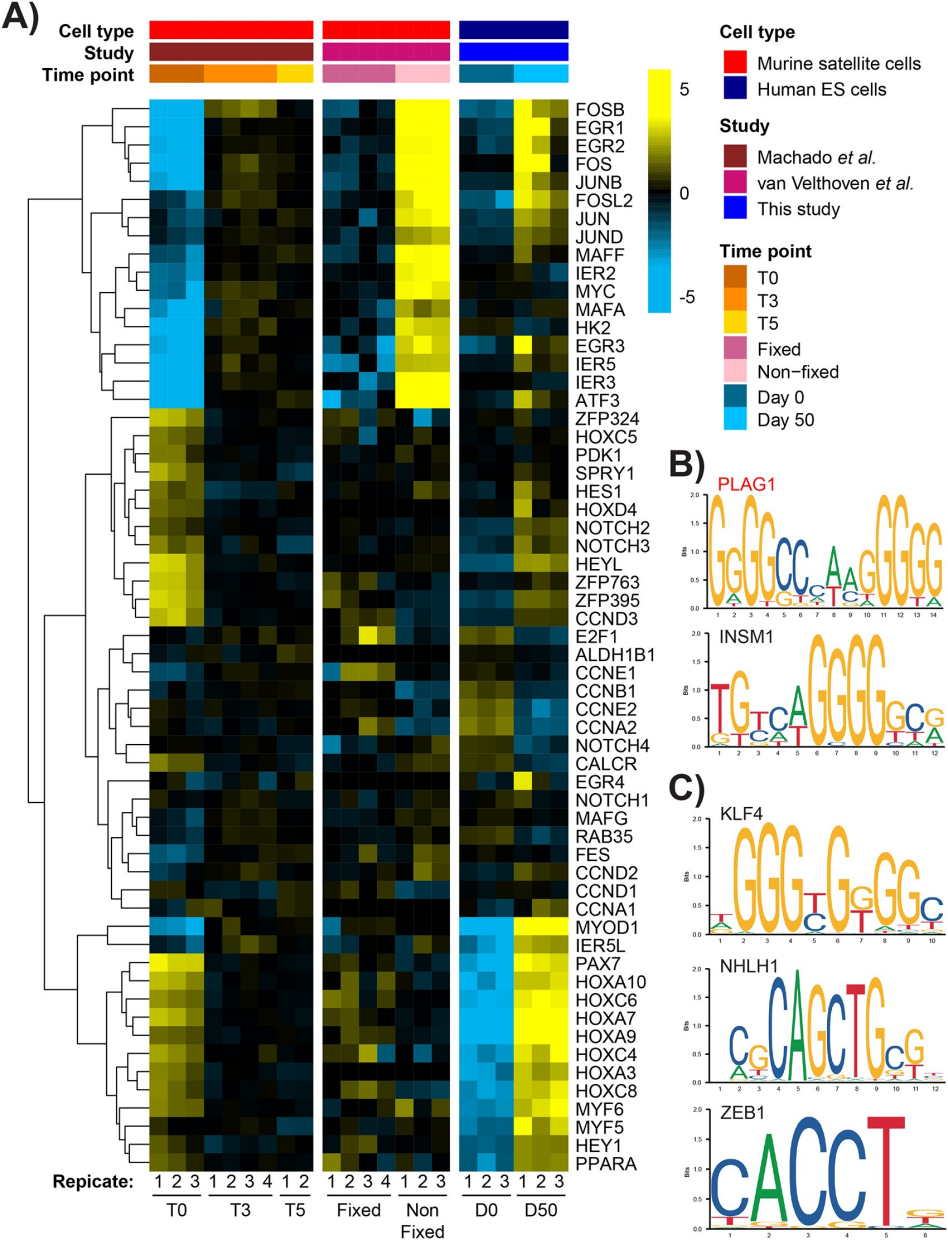
Day 50 cultures shared expression of previously considered quiescence markers, namely EGR and FOS/JUN genes. (A) A list of genes expressed in satellite cells from Fig 3D of Machado *et al*. 2017 was chosen to compare with the van Velthoven *et al*. 2017 study [72], and with day 50 hESC-derived myogenic cultures. Known quiescence genes SPRY1 and CALCR were also included. Genes (rows) were clustered based on their expression pattern across all studies. Analysis showed that day 50 cultures cluster more closely with quiescent cells (T0, Fixed) regarding NOTCH and HOX gene expression, but closer with early activated cells (T3, T5, Non-fixed) when comparing EGR and FOS/JUN gene families. The genes from Fig 3D of Machado *et al*. 2017 were also analyzed to identify significantly enriched TFBSs in genes that were more highly expressed in (B) quiescent, and (C) early activated satellite cells (Z > 5). The PLAG1 motif (red text) was significantly enriched in both quiescent and early activated gene sets.

Satellite cells can be susceptible to transcriptional changes in the several hours it takes to digest and release them from the host muscle, and subsequently FACS-isolate them [98]. Since merely processing quiescent satellite cells for study may initiate the early stages of activation, as in the Pallafacchina *et al*. and Liu *et al*. studies—we sought to assess the presence of the above NOTCH and FOS/JUN-related genes in cells more representative of the quiescent state, or true quiescence. Recently co-published studies by Machado *et al*. and van Velthoven *et al*. both prevented transcription *in situ* prior to satellite cell isolation to maintain their quiescence, termed T0 or Fixed, respectively [71,72]. Early activated satellite cells were obtained by isolating satellite cells from adult muscle without fixation, termed T3 or T5 in the Machado *et al*. study—representing 3 or 5 hours for isolation—or “Non-fixed” in the van Velthoven *et al*. study. These cells can be considered analogous to the “early activated cells” from the Pallafacchina and Liu studies (Fig 3A and 3B). The genes selected by Machado *et al*. in Fig 3D of their publication were used to compare day 50 cultures to true quiescent (T0 or Fixed) and early activated (T3 and T5 or Non-fixed) satellite cells of either the Machado *et al*. or van Velthoven *et al*. study, respectively (Fig 4A). Analysis showed that day 50 cultures cluster more closely with non-fixed early activated cells regarding EGR and FOS/JUN gene expression, but cluster more closely with T0 quiescent cells concerning NOTCH and HOX expression. TFBS analysis of the genes in Fig 4A that were more highly expressed in quiescent cells revealed the significant enrichment of binding sites also shared with the previous “Myotome / Limb Muscle” cluster analysis: PLAG1 and INSM1 (Fig 4B)(Table 2)(S2S Table). Genes more highly expressed in early activated cells also showed significant enrichment for PLAG1 sites, in addition to KLF4, NHLH1, and ZEB1 (Fig 4C)(S2T Table).

FOS/JUN heterodimers form the AP1 transcription factor complex; its role in either maintaining satellite cell quiescence or initiating differentiation may depend on the ratios of the various FOS and JUN subunits [80,81]. Regardless of AP1 function, however, the Machado *et al*. and Velthoven *et al*. datasets show a clear association between increased FOS/JUN gene expression and early activation, suggesting that expression of genes in the MAPK cascade may be more indicative of early activation than of quiescence. Other MAPK members like MAPK14 (p38a) are well established drivers of myogenic differentiation in the embryo and adult myoblasts [99–101].

The greater resolution between quiescence and activation provided by the Machado *et al*. and Velthoven *et al*. studies suggest that SMPs within day 50 cultures may exist in a state closer to early activation, or a heterogeneous mix of quiescent- and early activation-like states.

### Day 50 hESC-derived skeletal muscle is comparable to fetal muscle and isolated hESC-derived myoblasts from other studies

To build support for *bona fide* genetic markers and signatures of hESC-derived muscle, the gene expression profile of day 50 cultures was compared to other human ESC-derived skeletal muscle [24]. Choi *et al*. generated skeletal muscle by first inducing mesoderm with 3 μM CHIR99021 for 3 days, followed by the NOTCH inhibitor DAPT for 1 week. Myoblasts were FACS sorted for NCAM1^+^/HNK1^-^ at 30 days of differentiation, and the resulting cells were used for mRNA microarray analysis. Choi *et al*. also harvested mRNA from 18–19 week old fetal muscle for comparison. For clarity, the time points of the present study are referred to as days 0 and 50, and those of the Choi *et al*. study as days 0′ and 30′.

**Fig 5:**
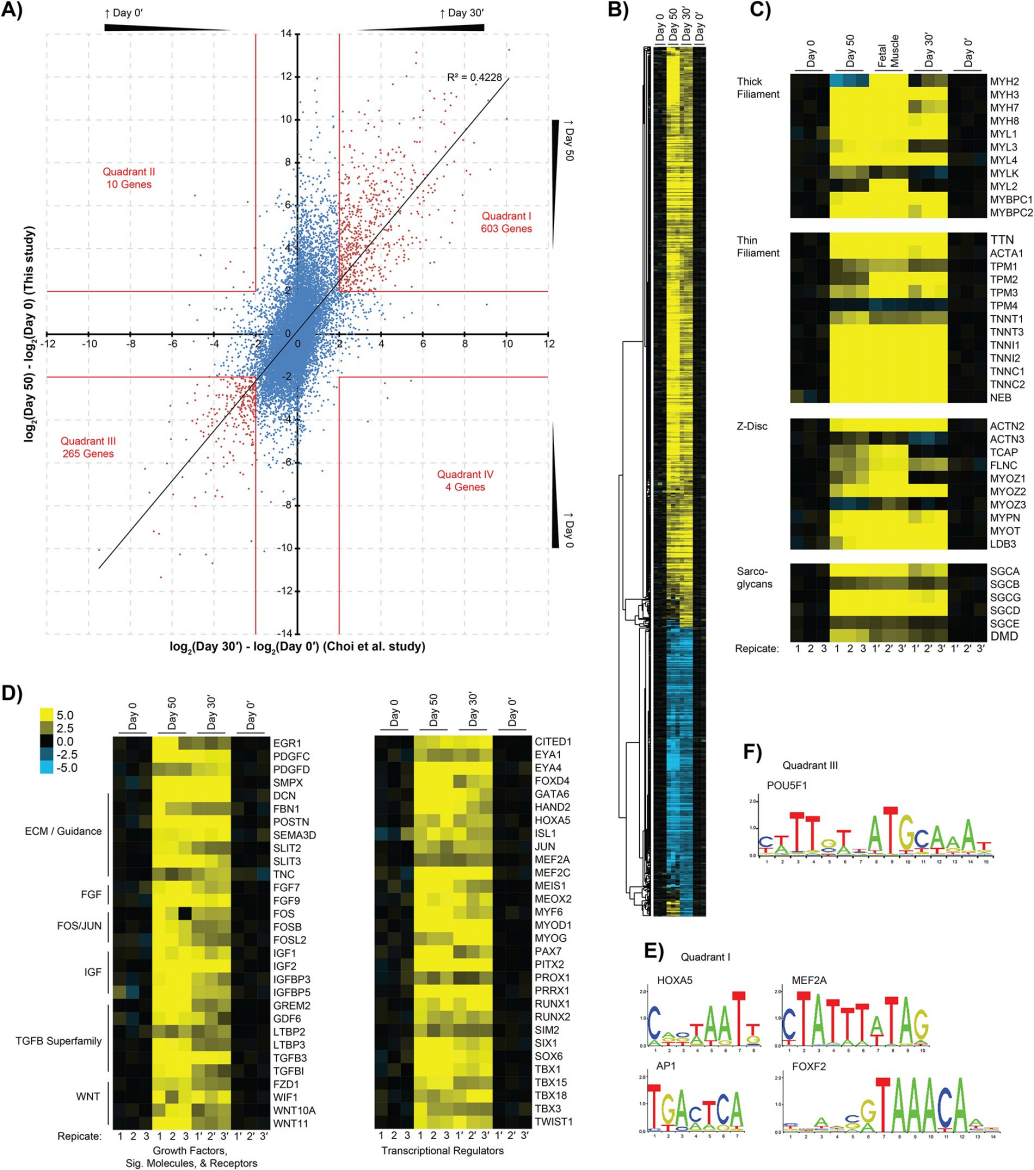
hESC-derived cultures show expression profiles resembling quiescent/early activated satellite cells, and novel/known SMP surface markers. (A) The mean expression of individual genes from [12] and [24] were standardized to the mean of their respective day 0 samples, and gene lists were generated from the four plot quadrants given an absolute 4-fold change cut-off. (B) Genes were filtered to select only those with at least 4-fold change in expression on either array relative to their respective day 0 samples, and a heat map of individual replicates showed fairly congruent expression profiles. The specific identities and expression values for each gene can be found in S4 Table. (C) A list of key sarcomeric structural genes was adapted from (85). Day 50 cultures exhibited more fetal-like expression levels of MYH7, MYL3, MYLK and several Z-disc genes, while day 30′ cells appeared to express the tropomyosins TPM2/TPM3 at levels more similar to fetal muscle. (D) Genes from quadrant I were separated using PANTHER to identify growth factors, signaling molecules, receptors, and transcriptional regulators. A heat map of individual replicates for select genes were shown for each condition. Day 50 and day 30′ cultures share overlapping gene expression similar to that of quiescent satellite cells, including IGF1, PAX7, FOS/JUN and TGFB-signaling genes. (E) Quadrant I and (F) quadrant III gene lists were also analyzed to identify significantly enriched TFBSs (Z > 5). Only motifs whose parent transcription factor was contained within the same quadrant were considered, given 2-fold change cut-off.

The day 50 and the Choi *et al*. day 30′ arrays were contrasted with their respective day 0 and day 0′ controls; a plot of all genes represented on both arrays revealed a positive correlation between both myogenic cultures (Fig 5A). A general overview of all genes with at least 4-fold absolute change in expression relative to day 0 or day 0′ controls revealed 995 genes with fairly similar expression patterns in both datasets (Fig 5B)(S4 Table).

The two cultures shared 603 elevated genes in quadrant I, and showed significant enrichment for GO terms like striated muscle tissue development, muscle contraction, and embryo development (Fig 5C and 5D)[45]. As expected, the 265 genes in quadrant III were enriched for categories related to the cell cycle and stem cell population maintenance. Expected myogenic transcription factors in quadrant I included the MRFs—with the exception of MYF5 which was only elevated in day 50 cultures—as well as MEF2A, MEF2C, PAX7, and SIX1. Also present were embryonic myogenesis genes EYA1, EYA4, and SOX6; limb bud genes MEOX2 and PRRX1; and several genes known to regulate craniofacial myogenesis ISL1, PITX2, SIM2, TBX1, and TWIST1 [102,103]. The craniofacial skeletal myosin MYH6 was also upregulated with both protocols and in fetal muscle [104]. Interestingly, PAX3 fell under quadrant II, showing elevated expression in day 50 cultures but reduced expression in day 30′ cells.

Overall, the sarcomeric gene expression seen with both protocols appeared similar to fetal muscle, although day 50 cultures appeared to have more fetal-like expression of thick filament and Z-disc genes, likely due to their longer differentiation period relative to the Choi *et al*. 30 day-old cultures (Fig 5C). Signaling pathways with a number of elevated genes included the TGFB superfamily, transcription factors downstream of the MAPK cascade, and IGF proteins: a similar expression profile to early activated satellite cells from Fig 3.

Interestingly, quadrant I contained surface markers of murine Lbx1^+^ embryonic muscle progenitors ITGA4 and its ligand VCAM1 (Table 5)[105–107], which also marks adult satellite cells or myotubes [3,8]. Quadrant I also showed surface markers previously used to enrich either adult satellite cells or embryonic SMPs, including CXCR4 [106], ERBB3 [18], NCAM1 [24,27], common satellite cell or developmental muscle markers ITGA7 and ITGB1 were absent as they were highly expressed throughout differentiation [6,108]. Other markers—CD82 [14,109], ICAM1 [74,110,111], and NGFR [18]—were only elevated in day 50 but not day 30′ cultures. This may be a result of the NCAM1^+^/B3GAT1^-^ (HNK1) FACS profile used by the Choi *et al*. study; NCAM1 may mark only a subpopulation of embryonic SMPs [18]. Also, over 60% of the Choi *et al*. NCAM1^+^/B3GAT1^-^ cells were already MYH^+^ indicating that PAX7^+^ SMPs may represent a smaller fraction of their cultures compared to day 50 cultures [12], skewing their gene expression profile towards mature muscle markers.

**Table 5:** Most surface proteins shared between day 50 and day 30ʹ cultures were also expressed in human fetal muscle.

Category	Total Genes in Category	Genes Shared with Fetal	Genes
Day 50 Only	272	49	A1BG, ACVRL1, ADRA1B, AGRN, **AGTR1**, AKT2, AMHR2, ANKS1B, ASB6, ATP7A, BAI2, BAI3, BDKRB1, BDKRB2, BEST1, BZRAP1, C6orf89, CACNA1C, CACNA1G, CACNA1H, CACNB2, CACNB3, **CACNG1**, **CACNG6**, CAMK1D, **CD36**, **CD97**, CDK19, CDKL2, CDON, CHRM1, CHRNA3, CHRNA4, CHRNG, CLEC4A, CLIC2, CLIC3, **CLIC5**, CLIC6, CMKLR1, CNGA3, CNR1, CNTFR, CNTN2, COL20A1, **COL6A2**, CRELD1, CTNNA3, CX3CR1, **DLK1**, DMBT1, DNAJC6, DRD4, EDNRA, **EGFLAM**, EGFR, ELFN2, EPHB2, EPHB3, EPOR, **EPS8**, EPS8L1, **ERBB4**, F2RL2, FAM89A, FAS, FBLN2, FBN2, **FCGRT**, FCRLB, **FGFRL1**, FLT3, FRZB, **FXYD1**, FXYD2, FXYD3, FXYD6, **FZD10**, **FZD4**, FZD6, GAB2, GABARAP, GABRA2, GABRB1, GABRR1, GCGR, GLRA2, GPR146, GPR153, GPR162, GPR179, GPR182, GPRASP1, GPRC5C, GRASP, GRID1, GRIK1, GRIK3, GRIN2D, GRIN3B, GRK4, **GRK5**, HTR2A, HTR2B, IFNAR1, IGFALS, IL10RA, IL10RB, IL18R1, IL1RAP, IL1RAPL2, **IL20RA**, IL2RG, IL6R, INSR, IRAK2, ITGB3, ITGB4, ITGB8, **ITPRIPL2**, KCNA5, KCNA7, KCNAB2, KCNAB3, KCNB1, KCNC1, KCNE2, KCNIP4, KCNJ12, KCNJ3, KCNJ4, KCNJ6, KCNK2, KCNK6, **KCNMB2**, KCNQ1, **KCNT1**, KDELR3, KREMEN2, LAMA3, LAMB1, **LAMB2**, LAMB3, LDLRAD1, LEPR, **LGALS1**, **LGALS3**, **LIFR**, LIN7B, LINGO2, **LPAR1**, LPAR5, LRIG3, LRP10, LRP1B, LRRC24, **LRRC32**, **LRRC3B**, LRRN4, LRRTM1, LTBP4, **LY96**, MADCAM1, MAGED1, MAL, MAPK4, MCOLN3, MEGF9, **MRC2**, MS4A10, MS4A15, NGFR, NPY1R, NPY2R, NPY6R, NR0B1, NR2F1, NR4A1, NR4A3, NTN1, **NTN4**, NTN5, NTNG1, NTNG2, NTRK1, NTRK3, OLFM3, **OLFML1**, OPN3, OR51E1, OR51E2, OR5I1, OR7D2, OSMR, **PDGFRB**, **PEAR1**, PINK1, PLA2R1, PNRC1, **PPARA**, **PPARG**, **PPARGC1A**, **PRKAA2**, **PRKACA**, PRKCB, PRKCE, PRLR, PTGER2, PTGIR, PTH1R, PTPN3, PTPRD, PTPRF, PTPRH, **PTPRM**, PTPRQ, **RAMP1**, **RARRES3**, RIPK3, RIPK4, ROR2, RORA, **RORC**, RTN4RL1, RXFP4, RXRG, **S1PR1**, S1PR3, SCARA3, SCARB2, SCN2B, SCN4B, SCN7A, SERINC1, SORCS2, SORCS3, SSC5D, SSR1,SSTR1, STON1, TAS1R3, TCP10, TCP11L2, **TF**, **TGFBR3**, **THRA**, THRB, TLR2, TLR5, TMEM130, TMEM176A, TMEM176B, TMEM38B, TMEM63C, TMEM8B, TNFRSF14, TRADD, TRIL, TRPV2, **TTYH2**, ULK2, VASN, VDR, VIPR2, VN1R1, CD59, CD82, CD86, CD99, ITGA3, ITGA8, **ITGA10**, ITGA11
Quadrant I	99	77	ACVR2A, **AGTR2**, **AHR**, AHRR, **ANTXR2**, **ATP2A1**, **ATP2A2**, ATP2B4, ATP7B, **BEST3**, **BMPR2**, **CACNA1S**, CACNA2D4, **CACNB1**, **CALCRL**, **CAV2**, **CAV3**, **CHRNA1**, **CHRNB1**, **CHRND**, **CLCN5**, CLSTN2, **CNRIP1**, **COL12A1**, **COL14A1**, **COL21A1**, **COL6A3**, **COL6A6**, CXCR4, **DDR2**, DSCAML1, EFEMP1, **EFEMP2**, **EPHA3**, **ERBB3**, **FBLN5**, **FBN1**, **FZD1**, **GHR**, **GPNMB**, GPR123, **GPR133**, **GRIA2**, GRIK2, GRIN2A, HIPK3, **IL11RA**, **IL1R1**, **IL6ST**, **ISLR**, **ITGB6**, **KCNE4**, KCNH2, **KCNJ2**, **KCNJ8**, **KCNMA1**, **KCNN3**, **KREMEN1**, **LAMA2**, LGALS2, LRRN3, **LTBP2**, **LTBP3**, **MATN2**, **MRAP2**, **NCAM1**, **NCOA1**, NCOA7, NPR3, NR2F2, **NR3C2**, NRBP2, **NTRK2**, **OLFML2A**, OLR1, **PDGFRA**, **PDGFRL**, **PMP22**, **POSTN**, PTGER3, **PTGER4**, **PTGFR**, **RAPSN**, **REEP1**, **RYR1**, **SCARA5**, **SCN3B**, **SPARC**, **SPARCL1**, **SRPK3**, **TGFBI**, **TMEM38A**, **VCAM1**, **CD44**, **CD109**, **CD302**, **ITGA4**, **GPR124**, **GPR126**
Day 30′ Only	18	11	**ANTXR1**, **CDK6**, CNTN4, CNTN6, **DLG2**, **GPR37**, IL13RA1, **LGR5**, **LRRC17**, MET, **NR3C1**, **OLFML2B**, **PTGFRN**, PTPRO, RARA, **RYR3**, SCN3A, **TGFBR2**

PANTHER was used to identify receptors, channels, and cell adhesion molecules within genes shared between day 50 and day 30ʹ cultures, or genes unique to either dataset. Several cluster of differentiation (CD), integrin, and G-protein coupled receptors not recognized by PANTHER were manually added. Genes that were also expressed in the Choi *et al*. fetal muscle sample were bolded. Several surface markers known to identify embryonic skeletal muscle progenitors or satellite cells—CXCR4, ERBB3, ICAM1, NCAM1, VCAM1—were shared between both hESC-derived cultures while some surface markers, however, were only elevated in Day 50 cultures: CD82 and NGFR.

The identification of many previously identified surface proteins suggests that the remaining list of shared receptors may contain additional uncharacterized SMP surface markers. For example, quadrant I contained adhesion G-protein coupled receptors with potential links to muscle and its development: ADGRA2 (GPR124), ADGRD1 (GPR133), and ADGRG6 (GPR126). One interesting candidate—ADGRG6—is highly expressed from E10.5 to E12.5 in the somitic mesoderm [112,113], it may mark the pre-myocyte stage of muscle development [113], and its expression was associated with DMD (Table 1), after the onset of PAX3 expression and prior to that of the MRFs. Also, the Pallafacchina *et al*. study showed ADGRG6 is expressed roughly 2-fold higher in early activated relative to late activated satellite cells [70].

ADGRG6 may be related to the ERBB pathway. ERBB1/2/3 mark murine satellite cells within 6 hours of activation, and mediate anti-apoptotic survival cues during muscle injury [114,115]. ERBB3 is a marker of PAX7-positive hPSC-derived SMPs [18], and its expression clusters with that of ADGRG6 and DMD in the present study (Table 1), suggesting ADGRG6 as an interesting candidate for further examination.

TFBS enrichment between day 50 and day 30′ cultures revealed significant enrichment of expected TFBS for HOXA5, and MEF2A within quadrant I (Fig 5E)(S2U Table). Other HOX family proteins with similar homeodomain binding sequences to HOXA5 may be transcriptionally active in both cultures. Indeed, both cultures show elevated expression of 12 HOX genes, including HOXA10 and HOXA11, which are associated with limb development [116], and FOXF2. While the latter has no clear role in skeletal muscle, its consensus sequence may be present in the results due to its similarity to other expressed forkhead family factors [117], which have known roles in energy metabolism and autophagy in muscle cells [93,118–122]. POU5F1 TFBSs were associated with genes highest in pluripotent day 0 and day 0′ cultures as expected (Fig 5F)(S2V Table).

To further explore the similarities between our dataset and other published sets, we examined a subset of transcription factors from Table 2, which were expressed in clusters 1–4 and had TFBS enriched in the same cluster or the next, and compared this to day 30′ cultures from Choi *et al*. (S3 Fig). We also compared this dataset to a time course of differentiation described by Wu *et al*., using PAX7-dsTomato and MYF5-GFP to enrich cultures on day 4 and day 10, respectively [123]. The day 10 MYF5^+^ cultures, termed D10P, were differentiated further and harvested on day 20, termed Diff. There was overall agreement in gene expression changes induced by our differentiation protocol and those of Wu *et al*. and Choi *et al*. Furthermore, we identified genes showing an 8-fold or greater change in their expression between ESCs and day 50 of our protocol, with adjusted p-value < 0.01, and analyzed their expression in the Wu *et al*. and Choi *et al*. datasets (S4 Fig). The results of this heat map indicate that general trends in gene expression changes are conserved. This is also reflected by high Pearson correlations in log_2_ fold changes: 0.62 and 0.76 between this study (day 50 versus day 0 hESCs) and Wu *et al*. (MYF5^+^ sorted cells versus day 0 hESCs) and Choi *et al*. (NCAM^+^/HNK1^-^ sorted cells versus day 0 hESCs), respectively. Therefore, despite differences in the protocols and the extent of PAX3/PAX7 expression, the general trends are similar, supporting the validity of our study.

Overall, the comparison of expression profiles from variously sourced hESC-derived skeletal muscle allows for the better understanding of the muscle tissue generated by these *in vitro* differentiation protocols. While overall similarities exist between the two protocols analyzed, day 50 cultures contain a greater number of known and potential surface markers for PSC-derived SMPs.

## Conclusions

**Fig 6:**
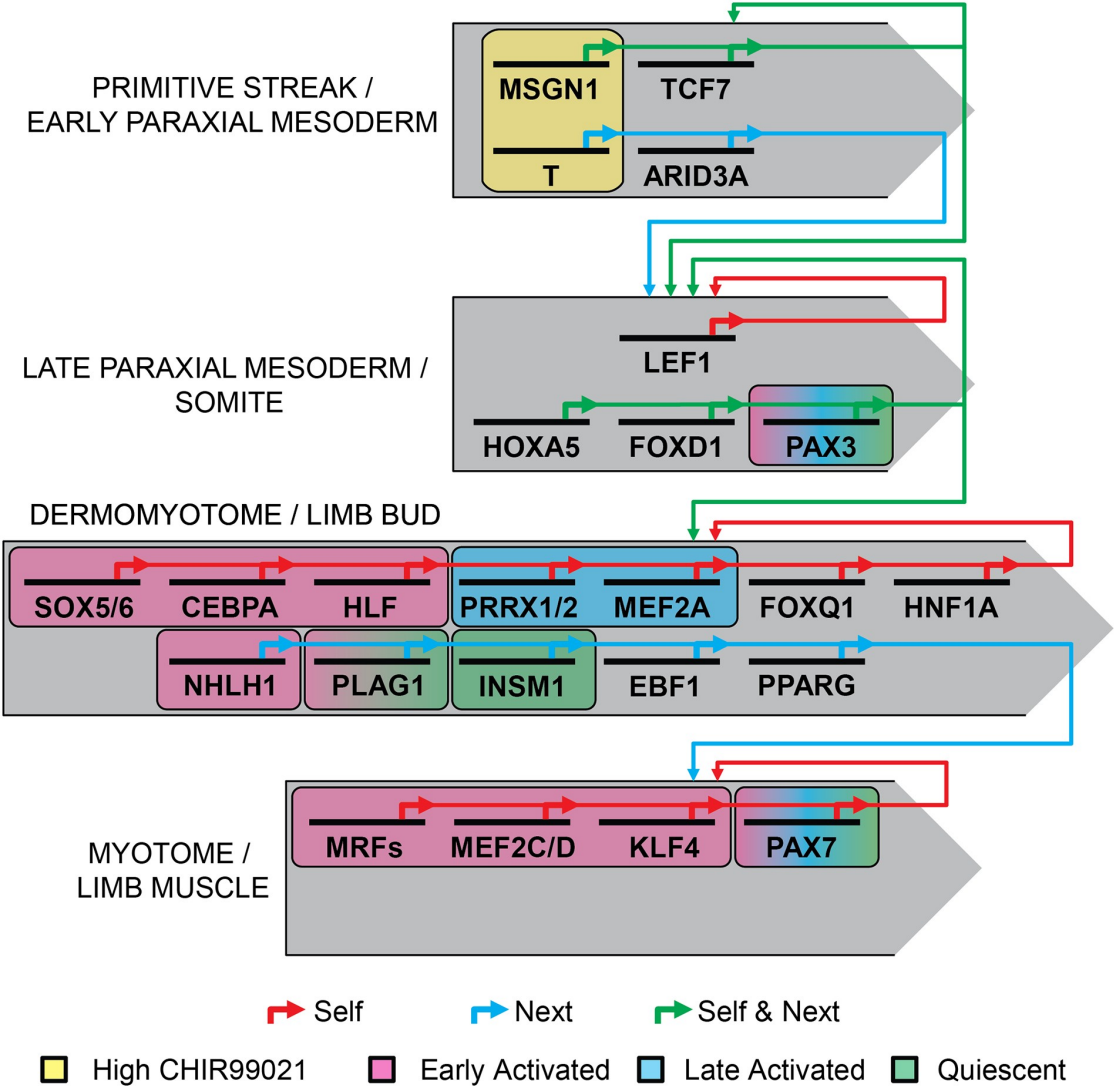
Putative cascade of transcription factors that regulated four stages of *in vitro* skeletal muscle development. Key transcription factors were taken from the TFBS analysis in Table 2; only motifs whose parent transcription factor was contained within the same or in the previous cluster were included. The colored arrows indicate whether a transcription factor may be regulating genes within its own cluster (red arrow), within the subsequent cluster (blue arrow), or both (green arrow) according to TFBS enrichment analysis. The significantly enriched TAL1::TCF3 motifs in the two earliest clusters have been manually attributed to MSGN1, owing to the two sites’ similarity [39]. Factors more notably expressed in cultures differentiated with high versus low CHIR99021 concentrations—T and MSNG1—are outlined (yellow box). Significantly enriched TFBSs in the promoters of genes shared between hESC-derived cultures and various satellite cell gene sets are also outlined: early activated (red box), late activated (blue box), and quiescent (green box). Hypergeometric probability analysis determined significant enrichment of PAX3/7 Soleimani *et al*. and Lilja *et al*. target genes in all 3 satellite cell datasets.

This study aimed to generate and analyze gene expression profiles of hESCs undergoing directed *in vitro* skeletal myogenesis [12]. Clustering of genes with similar expression patterns across the 50-day differentiation revealed 4 waves of gene expression related to myogenesis, designated Primitive Streak / Early Paraxial Mesoderm, Late Paraxial Mesoderm / Somite, Dermomyotome / Limb Bud, and Myotome / Limb Muscle stages. To summarize our in silico findings (Fig 6), a putative cascade of transcription factors can be discerned by determining the TFBSs that are significantly enriched in the promoter regions of the genes in each cluster and identifying which corresponding TFs are expressed in the same or the previous cluster shown in Table 2. Of these key transcription factors, T and MSGN1 staining or expression were greater with high—relative to low—CHIR99021 treatment (Fig 2A and 2D), 8 TFBSs were associated with early satellite cell activation (Figs 3E and 4C), 2 with late activated satellite cells (Fig 3F), and 2 with the quiescent satellite cell gene set (Fig 4B). While this model is hypothetical, the observation that the expected known factors were identified by this analysis provides validity to the approach and suggests that the accompanying previously unstudied factors may be novel myogenic regulators worthy of study.

A direct comparison between day 2 cultures differentiated with high- versus low-concentrations of CHIR99021 revealed that high CHIR99021-treated cultures had significantly greater expression of MSGN1 and TGFB signaling genes, including NODAL. It remains unclear what benefits exogenous inhibition of the TGFB superfamily from days 2–4 may provide to the high CHIR99021 approach [25], and if exogenous inhibitors that are sometimes used following CHIR99021 treatment in other differentiation protocols are compensating for CER1 or acting independently of it [23,25,38].

Comparing hESC-derived myogenic cultures to the gene expression profiles of murine satellite cells showed a greater similarity between day 50 and more quiescent-like or early activated rather than late activated satellite cells; this was underscored by the shared expression of quiescence-promoting or early activation genes in CEBP, FOS/JUN, NOTCH, and TGFB signaling pathways. Comparison to satellite cells identifies ITGA8 as a potential surface marker to isolate more early activated-like cells, and ITGB6 to potentially select out late activated cells. Lastly, expression of sarcomere-related genes appeared comparable between Shelton *et al*. and the Choi *et al*. hESC-derived myogenic cultures [24], but cultures did not yet express MYH2 and MYL2 as seen with 18-week human fetal muscle. Both approaches identified known surface markers for human pre-natal SMPs—CXCR4 [106], ERBB3 [18], and NCAM1 [27,124]—and highlight new G-protein coupled receptors ADGRA2, ADGRD1, and ADGRG6 that may help increase the resolution by which these SMPs can be defined or segregated.

Limitations of this study include the minimum number of time points analyzed and the use of unsorted bulk cultures. The latter makes it impossible to delineate gene expression in PAX7^+^ SMPs from that of myoblasts or myocytes, although it also ensures no populations are lost during sorting. A previous study achieved up to 96% PAX7^+^/PAX3^+^ SMP purity by FACS for B3GAT1^-^/CHRNA^-^/CXCR4^+^/MET^+^ in hESCs undergoing directed skeletal myogenesis, although this population was transitory [20]. Subsequently, it was shown that ERBB3^+^/NGFR^+^ can select for hESC-derived PAX3^+^/PAX7^+^ SMPs that appear better suited for long-term engraftment *in vivo* than unsorted cultures [18]. ADGRG6 was identified in the present study as a potential SMP marker that showed a similar expression pattern to ERBB3 and NGFR in these cultures. Therefore, it would be worthwhile to determine what SMP population—if any—ADGRG6 can identify during *in vitro* skeletal myogenesis.

Uncovering additional novel surface proteins to mark and isolate embryonic SMPs—used individually or in conjunction with previously established surface markers—may further increase the resolution by which researchers define different SMP populations, or the purity of their sorting. Given what we know about HOX clusters [125–127], it is possible that the pattern of HOX genes found in SMPs may provide a fingerprint regulating the future identity of the muscle they create. While other studies show *in vivo* transplantation of SMPs derived from the directed differentiation of hESCs or iPSCs, the possible effects of the donor cells’ developmental identity with regards to the transplantation site have not been well established [19,24,74,128]. Furthermore, a longer list of surface proteins to mark SMPs provides greater flexibility in choosing appropriate antibodies when staining or sorting myogenic cultures.

Ultimately, 50-day cultures contain a persistent PAX7^+^ SMP population with an expression profile of signaling pathways and transcriptional regulators similar to quiescent/early activated satellite cells, which could prove a promising source of material for stem cell therapy. Thus the Shelton *et al*. protocol stands in contrast with other *in vitro* differentiation protocols that have low percentages of—or gradually lose—PAX7 expressing SMPs as myotubes dominate [19,22–24]. However, a delicate balance must be struck between maintaining hPSC-derived SMPs in a more quiescent satellite cell-like state and not repressing their differentiation too heavily to the point that they no longer respond to physiological cues to differentiate. This study confirms the expression of many previously outlined SMP surface markers in 50-day cultures and identifies novel markers as an additional avenue to purify the persistent PAX7^+^ SMPs for future studies leading to potential stem cell therapies.

## Materials and methods

### Cell culture

Human embryonic stem cells (H9) were maintained and differentiated as in [13].

### Cell culture analysis

Phase-contrast microscopy, immunofluorescence staining, and qPCR analysis were performed as in [12]. Cell counting for Hoechst (Sigma–Aldrich, St. Louis, USA, B-2883) and T (Abcam, Toronto, Canada, AB20680) nuclear staining was performed as in [129]. Primary antibodies against PAX7 (Developmental Studies Hybridoma Bank, Iowa City, USA, AB528428), MKI67 (KI67)(Abcam, AB15580), MYOD1 (Abcam, AB133627), and secondary Cy3 goat anti-rabbit IgG (Jackson ImmunoResearch, West Grove, USA, 111-165-003), AlexaFluor546 goat anti-mouse IgG1 (ThermoScientific, Waltham, USA, A21123), and AlexaFluor647 goat anti-rabbit IgG (ThermoScientific, A21244) antibodies were also used. The antibody staining for PAX7, MYOD1, and KI67 were conducted similar to [13], with the exceptions that permeabilization was carried out with 0.5% TritionX-100 and 100 mM glycine in Tris-buffered saline (TBS), blocking solution was 2% bovine serum albumin (BSA), 5% goat serum, and 0.1% Tween20 in TBS, and the stains were mounted with PermaFluor (ThermoScientific, TA-006-FM). CellProfiler 3.0 was used to analyze the PAX7, MYOD1, and KI67 staining.

### Microarray gene expression profiling

Gene expression analyses were carried out in triplicate using three biological replicates of the directed differentiation protocol. Total RNA was purified using Total RNA Mini Kit (FroggaBio). A total of 40 ng of RNA was processed and fluorescently labeled using Agilent Low Input Quick Amp Labeling Kit (Agilent Technologies, Santa Clara, CA, USA). A total of 600 ng of Cy3-labelled cRNA was hybridized to Agilent 8x60 K-Human Genome Microarrays using Agilent Gene Expression Hybridization Kit. The microarrays were read on the Agilent DNA Microarray SureScan Scanner. The raw reads were filtered using a custom-made Perl script to retain only probes detected above background in at least 3 of the 24 samples. Probes were log_2_() transformed and quartile-normalized using Expander 7.0 software [30]. The data can be found online in the GEO repository (GSE131125).

### Clustering and significant gene list generation

Expander 7.0 was used to perform CLICK clustering and t-test statistical analysis [30,130]. Only probes with a difference of at least 2 in their log_2_() expression values, and absolute log_2_() expression of at least 5, were retained and used in clustering. Clustering was performed given a 0.85 homogeneity value, and the resulting clusters were manually grouped based on expression pattern similarity where deemed appropriate (S1 Table). T-test statistical analysis were performed in Expander 7.0 using probes that changed by at least 4-fold at the time point under investigation relative to day 0 and had at least 5 log_2_() expression in at least one sample. Unless otherwise stated, p ≤ 0.05 and FDR ≤ 0.05 (by the Benjamini-Hochberg method) were used for Expander 7.0 statistical analysis. One-way ANOVA (p < 0.05) and post-hoc Tukey test (p < 0.05) were used to analyze the quantified immunofluorescence and qPCR results.

### Gene list analysis

The ToppGene Suite was used to identify significantly enriched Molecular Function and Biological Process Gene Ontology categories within a given gene set (p ≤ 0.05, q ≤ 0.05 by the Bonferroni method)[31]. Representative Gene Ontology categories and example genes or gene families were manually chosen from results with q ≤ 0.05 by Bonferroni correction. PANTHER Classification System was used to identify genes from a given set that were classified as growth factors, receptors, signaling molecules, or transcription factors [131]. Genes unclassified by PANTHER were manually labeled where deemed appropriate based on published knowledge. oPOSSUM 3.0 was used to identify transcription factors with significantly over-represented binding sites within a given gene set, using a conservation cut-off = 0.60 and considering 2 000 base pairs of upstream and 2 000 base pairs downstream sequence from transcription start sites [132](S2 Table). Only factors with Z-score ≥ 5 were considered significantly enriched. Heatmaps were generated using Java TreeView 3.0 software [133].

### Gene expression analysis with published datasets

The mRNA microarrays used for comparison to day 50 myogenic cultures correspond to *in vitro* activated mouse satellite cells (GSE15155)[70], *in vivo* activated mouse satellite cells (GSE47177)[8], and hESC-derived and fetal skeletal muscle (GSE70955)[24].

With the Shelton *et al*. dataset, the log_2_-fold changes in probe expression were calculated for each day 50 replicate by subtracting the average of triplicate undifferentiated day 0 values. Similarly, fold changes were calculated for GSE70955 skeletal muscle replicates by subtracting the average of triplicate undifferentiated cell samples. The log_2_-fold changes for probes from GSE15155 and GSE47177 were calculated by subtracting the average of triplicate activated satellite cell samples from each quiescent satellite cell sample. In the event where multiple probes exist for a single gene, only the probe with the greatest absolute fold change was considered for further analysis. Genes were paired between arrays according to HUGO Gene Nomenclature Committee (HGNC) gene symbol identity. Mouse specific genes with no human counterpart were excluded from each list.

Day 50 log_2_ fold changes for each gene were plotted against those of the three comparative arrays; genes were divided into four quadrants—given a 4-fold cut off—in order to find commonly or differentially expressed genes between the datasets.

## Supporting information

S1 Table Complete list of gene identities from clustering analysis. (XLSX)

S2 Table Full output of TFBS analysis results. (A-L) Related to Table 2. (M-R) Related to Fig 3E–3G. (S and T) Related to Fig 4B and 4C. (U and V) Related to Fig 5E and 5F.
(XLSX)

S3 Table List of microarray probes with Pearson correlation coefficient with NODAL of 0.8 or higher. (XLSX)

S4 Table Complete list of genes with at least absolute 4-fold differential expression in Day 50 or Day 30′ cultures relative to Day 0 or Day 0′, respectively. (XLSX)

S1 Fig Gene set enrichment analysis indicates EGF response is upregulated in high- versus low-CHIR99021 samples. Gene set enrichment analysis (GSEA) was performed using the MSigDB collection of gene sets using as phenotypes “Day 2 High CHIR” and “Day 2 Low CHIR”. For statistical analysis, permutation was done on gene sets because the number of samples per condition is too small for sample permutation to be reliable. The heat map represents median-centered log_2_() expression in hESCs and in day 2 samples.
(TIF)

S2 Fig Detection of Pax7-expressing cells with features of quiescent satellite cells. Immunostaining was performed on cultures from day 32 or day 46 with antibodies against PAX7 and either MYOD1 or the proliferation marker KI67, and secondary antibodies coupled to Cy3 or Cy5. DNA was counterstained using DAPI. Images were acquired and analyzed using CellProfiler 3.0 [134], where nuclei positive for PAX7 and/or the other marker (MYOD1 or KI67) were identified. The fluorescence signal was smoothed—to erase hot-pixels from the CCD camera—and rescaled to spread the entire range of possible intensity values, and the median pixel signal intensity in each nucleus was calculated for each color channel. These per-cell intensity measurements were plotted in Microsoft Excel.
(TIF)

S3 Fig Overall agreement in gene expression changes induced by our differentiation protocol and two other approaches. The expression of genes listed in our model figure (Fig 6) in our dataset and those reported by Wu *et al*. [123] and Choi *et al*. [24]. Expression in each sample is centered on the median of the samples of the respective study.
(TIF)

S4 Fig The agreement between studies in S3 Fig is generalized. Genes showing an 8-fold or greater change in their expression between day 0 hESCs and day 50 of our protocol, with adjusted p < 0.01, were identified, and their expression in the Wu *et al*. [123] and Choi *et al*. [24] datasets were analyzed.
(TIF)
