# Supplementary figures and images for: Gene expression profiling of skeletal myogenesis in human embryonic stem cells reveals a potential cascade of transcription factors regulating stages of myogenesis, including quiescent/activated satellite cell-like gene expression
Source: PLoS One. 2019 Sep 27;14(9):e0222946. doi: 10.1371/journal.pone.0222946 (PMC6764674; doi:10.1371/journal.pone.0222946)

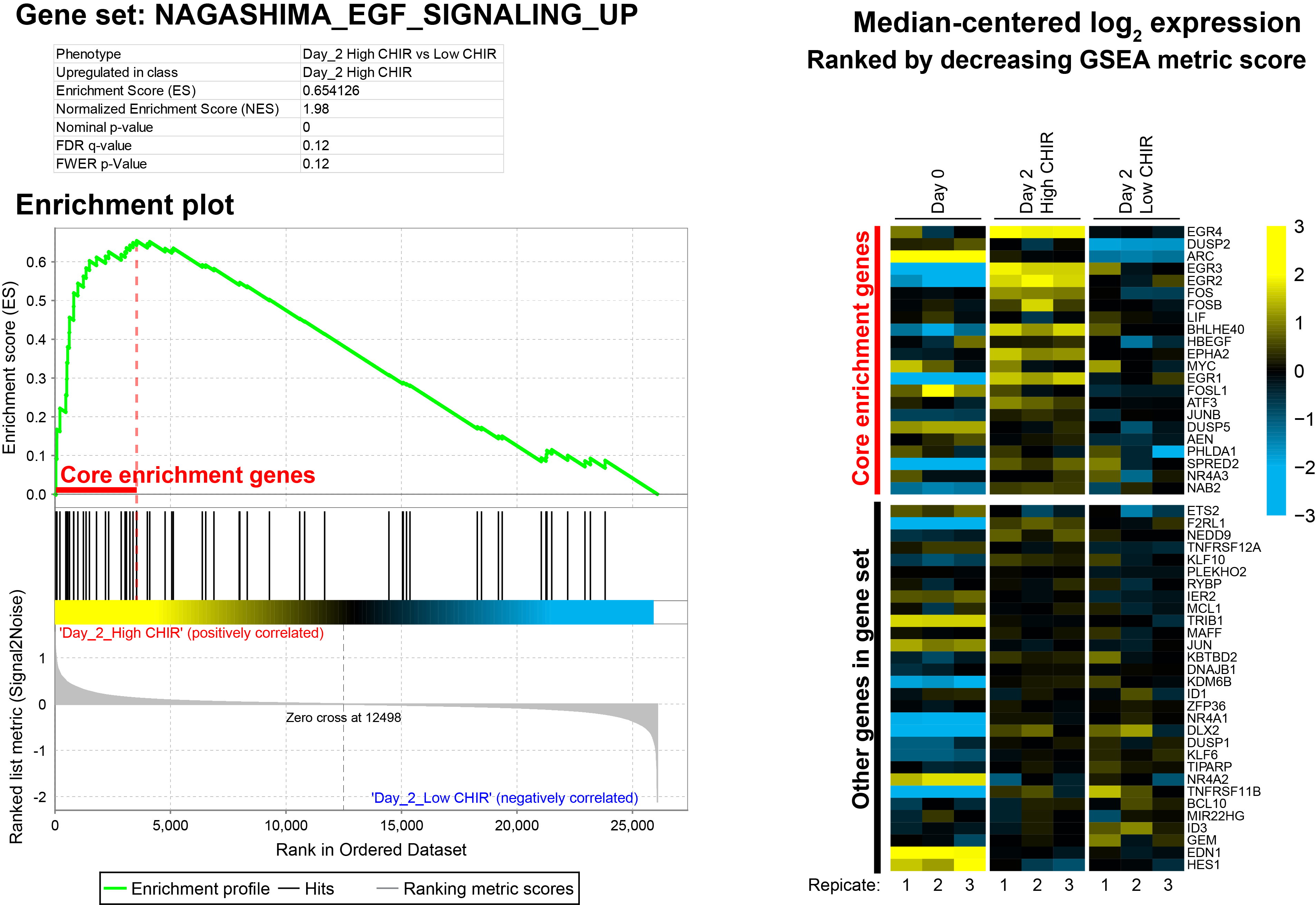

Supplement: S1 Fig — Gene set enrichment analysis (GSEA) was performed using the MSigDB collection of gene sets using as phenotypes “Day 2 High CHIR” and “Day 2 Low CHIR”. For statistical analysis, permutation was done on gene sets because the number of samples per condition is too small for sample permutation to be reliable. The heat map represents median-centered log2() expression in hESCs and in day 2 samples. (TIF) [file pone.0222946.s005.tif]

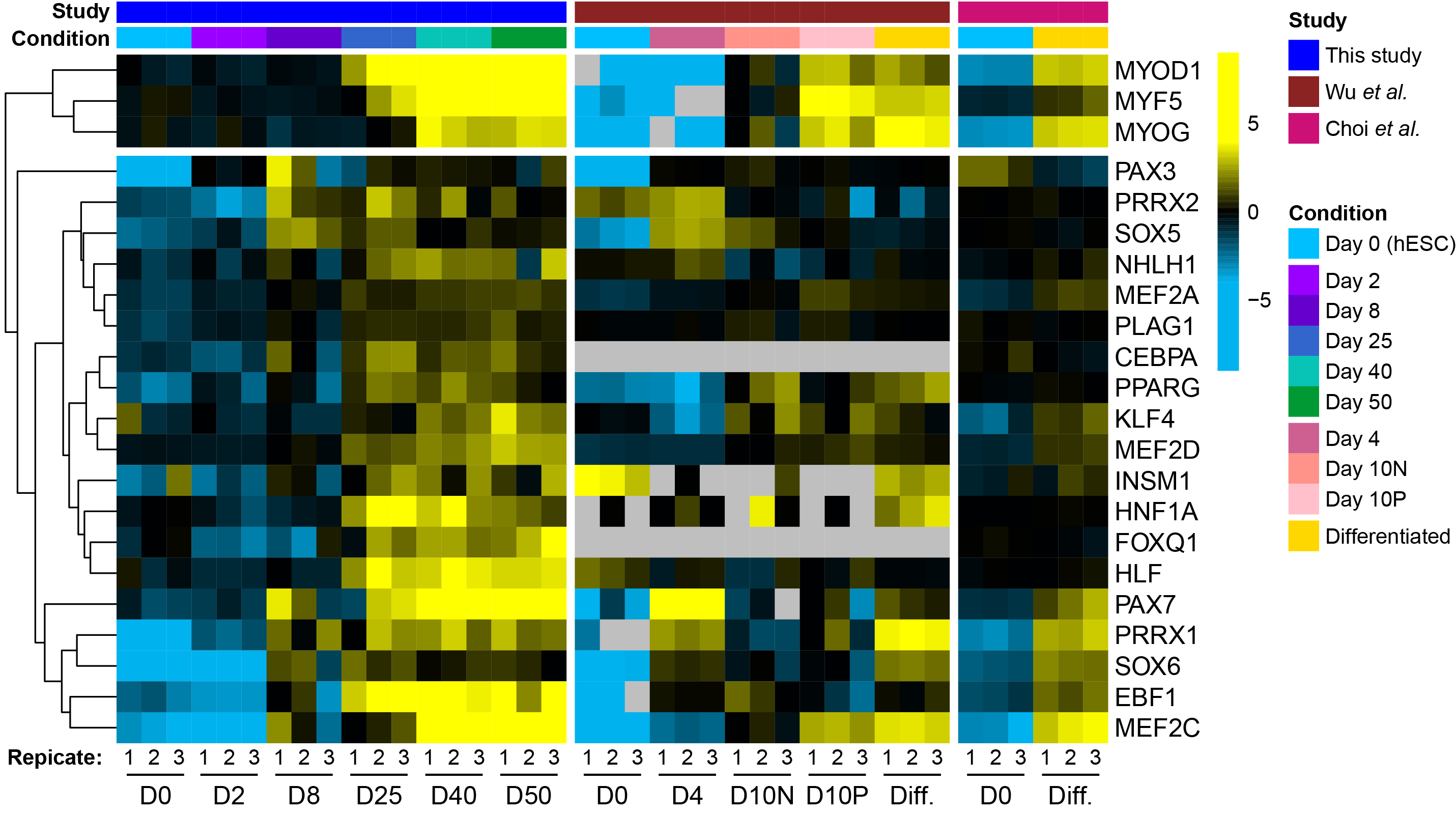

Supplement: S3 Fig — The expression of genes listed in our model figure (Fig 6) in our dataset and those reported by Wu et al. [123] and Choi et al. [24]. Expression in each sample is centered on the median of the samples of the respective study. (TIF) [file pone.0222946.s007.tif]

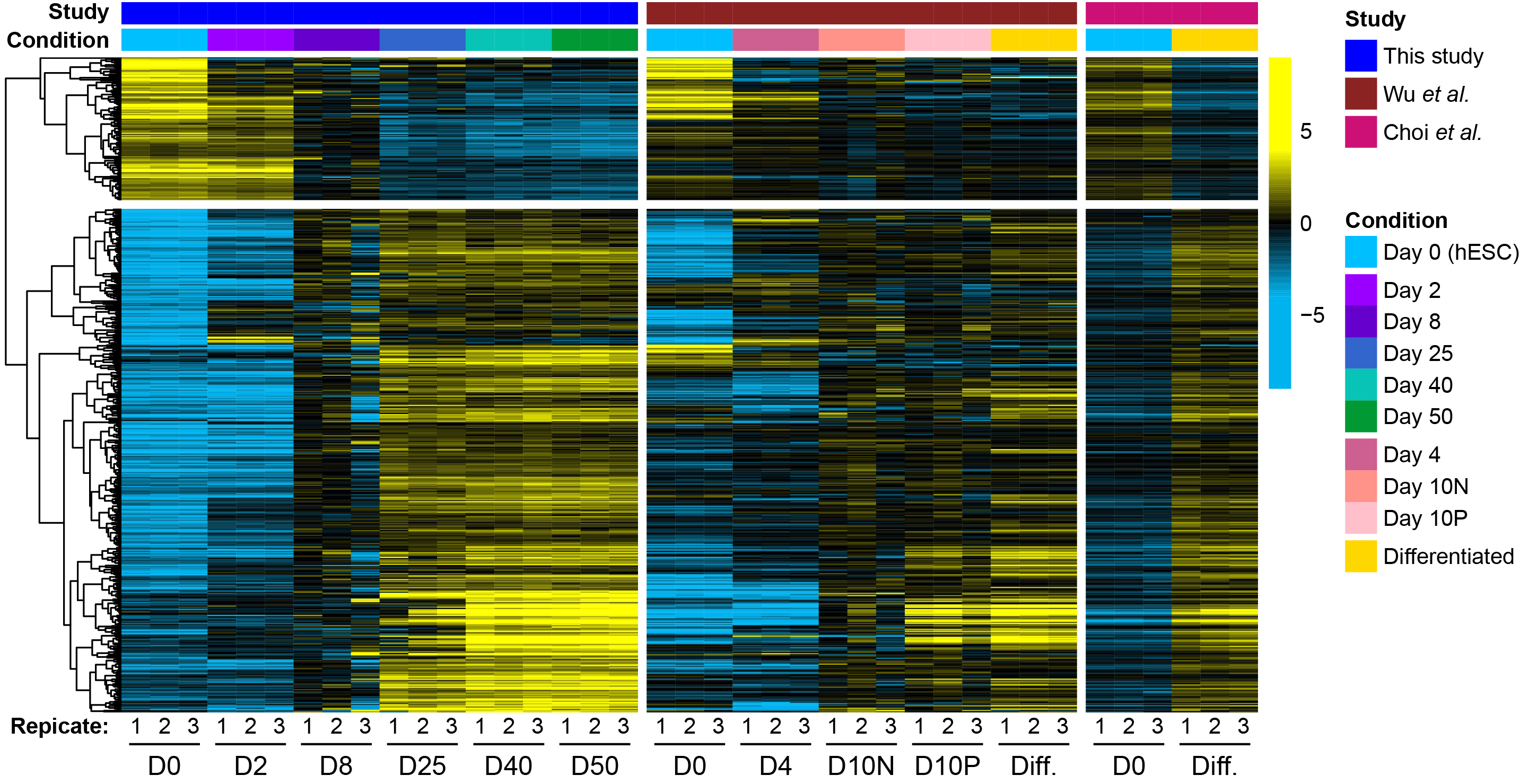

Supplement: S4 Fig — Genes showing an 8-fold or greater change in their expression between day 0 hESCs and day 50 of our protocol, with adjusted p < 0.01, were identified, and their expression in the Wu et al. [123] and Choi et al. [24] datasets were analyzed. (TIF) [file pone.0222946.s008.tif]
